# Using a thermal gradient table to study plant temperature signalling and response across a temperature spectrum

**DOI:** 10.1186/s13007-024-01230-2

**Published:** 2024-07-29

**Authors:** Myrthe Praat, Zhang Jiang, Joe Earle, Sjef Smeekens, Martijn van Zanten

**Affiliations:** 1 Plant Stress Resilience, Institute of Environmental Biology, Utrecht University, Padualaan 8, Utrecht, 3584CH The Netherlands; 2 Molecular Plant Physiology, Institute of Environmental Biology, Utrecht University, Padualaan 8, Utrecht, 3584CH The Netherlands; 3 Netherlands Plant Eco-Phenotyping Centre, Institute of Environmental Biology, Utrecht University, Padualaan 6, Utrecht, 3584CH The Netherlands; 4 Present address: Evolutionary Plant Ecophysiology, Groningen Institute for Evolutionary Life Sciences, University of Groningen, Nijenborgh 7, Groningen, 9747AG The Netherlands

**Keywords:** Thermomorphogenesis, Thermal gradient table, Cold acclimation, Temperature, Dose-response, Arabidopsis, Lettuce, Tomato

## Abstract

Plants must cope with ever-changing temperature conditions in their environment. In many plant species, suboptimal high and low temperatures can induce adaptive mechanisms that allow optimal performance. Thermomorphogenesis is the acclimation to high ambient temperature, whereas cold acclimation refers to the acquisition of cold tolerance following a period of low temperatures. The molecular mechanisms underlying thermomorphogenesis and cold acclimation are increasingly well understood but neither signalling components that have an apparent role in acclimation to both cold and warmth, nor factors determining dose-responsiveness, are currently well defined. This can be explained in part by practical limitations, as applying temperature gradients requires the use of multiple growth conditions simultaneously, usually unavailable in research laboratories. Here we demonstrate that commercially available thermal gradient tables can be used to grow and assess plants over a defined and adjustable steep temperature gradient within one experiment. We describe technical and thermodynamic aspects and provide considerations for plant growth and treatment. We show that plants display the expected morphological, physiological, developmental and molecular responses that are typically associated with high temperature and cold acclimation. This includes temperature dose-response effects on seed germination, hypocotyl elongation, leaf development, hyponasty, rosette growth, temperature marker gene expression, stomatal conductance, chlorophyll content, ion leakage and hydrogen peroxide levels. In conclusion, thermal gradient table systems enable standardized and predictable environments to study plant responses to varying temperature regimes and can be swiftly implemented in research on temperature signalling and response.

**Supplementary Information:**

The online version contains supplementary material available at 10.1186/s13007-024-01230-2.

## Background

Ambient temperature is an important environmental cue that fluctuates momentaneous, diurnally and seasonally. Temperature is a physical entity that can be seen as a gradient that ranges from freezing to heat in the so called ‘physiological’ range [1–4]. The perceived temperature provides plants with valuable information to tune growth, development and phenology to its current (temperature) environment. Although extreme, i.e. stressful, temperatures can cause damage to plants and generally leads to growth cessation, or even death, acclimation of plant architecture, physiology, phenology, growth and development to mild sub-optimal cold and warm temperatures can help plants to maintain optimal performance under unfavourable (sub-optimal) temperature conditions [5–7].

Current climate change leads to more extreme and irregular weather events and increased levels of stressful temperatures, exposing ecosystems and agriculture to episodes of severe drought, flooding and heat [8–11]. Independent methods consistently project climate change to have a negative impact on crop yield and already a 1 °C increase in average global temperature is projected to lead to major yield losses in staple crops such as wheat, maize and rice [12–16]. These adverse effects coincide with an increase in global food and feed demand and poses a considerable challenge for the agricultural sector to increase food security [13, 17]. Thus, there is a pressing need for the development of thermotolerant crops that can withstand adverse temperatures.

### Thermomorphogenesis; acclimation to mild warm temperatures

Acclimation to high ambient temperatures, as for instance seen in the model plant *Arabidopsis thaliana* when grown at 27–28 °C compared to standard laboratory conditions (20–22 °C), is called thermomorphogenesis [2, 18–20]. In Arabidopsis seedlings, thermomorphogenesis is characterized by elongated hypocotyls and hyponasty (upward movement) of the cotyledons [18, 21, 22]. In adult vegetative plants, high ambient temperatures result among other traits in longer petioles, hyponasty and alterations in leaf blade shape and size [5, 22, 23]. Together, thermomorphogenesis triggers an open rosette structure that is proposed to aid the plant’s cooling capacity [24, 25, 26]. High ambient temperature also leads to early flowering (reviewed in [27]). In addition to Arabidopsis, thermomorphogenesis occurs in many crop species such as tomato, wheat and cabbages [16, 19].

The transcription factor PHYTOCHROME INTERACTING FACTOR 4 (PIF4) is considered a central signalling hub for thermomorphogenesis [18, 28]. In response to high temperature, PIF4 levels rise and the protein binds to the promoters of auxin biosynthesis genes such as *YUCCA8*, thereby enhancing auxin biosynthesis at high temperatures, leading to thermomorphogenesis [18, 29]. Accordingly, *pif4* null mutants lack the ability of e.g. hypocotyl elongation in response to a higher ambient temperature [30]. Another molecular maker for high temperature is *HEAT SHOCK PROTEIN 70* (*HSP70*) as its transcription directly scales with the perceived temperature [31].

In *Arabidopsis thaliana* so far three *bona fide* thermosensory events have been described involving phytochrome B (phyB) [32], EARLY FLOWERING 3 (ELF3) [33] and PHYTOCHROME INTERACTING FACTOR 7 (PIF7) [34]. Likely, more sensory events remain to be discovered. Warm temperature-dependent conversion of active phyB Pfr to the inactive Pr conformation leads to nuclear exclusion of phyB and release of PIF4 inhibition [29, 32, 35]. EARLY FLOWERING 3 (ELF3) is a component of the evening complex (EC) of the circadian clock that restricts *PIF4* expression during the early evening [36]. Additionally, ELF3 can interact with PIF4 protein in an EC-independent manner, preventing PIF4 to activate its targets [37]. Later, it was shown that the prion-like domain of the ELF3 protein provides thermosensory input [33]. The prion-like domain causes ELF3 to form reversible aggregates by liquid-liquid phase separation at high ambient temperatures [33]. As a result, ELF3 can no longer be integrated into the evening complex, nor can function as a negative regulator of *PIF4*. Interestingly, temperature sensitivity of ELF3 scales with the length of the polyQ tract in the domain [33]. In the same year, it was shown that (PHYTOCHROME INTERACTING FACTOR 7) PIF7 plays an important role in regulating growth during daytime. It was found that translation of *PIF7* mRNA, and thereby protein levels, are enhanced by high temperature-dependent relaxation of a *PIF7* mRNA hairpin structure [34]. Alike PIF4, PIF7 can bind to promoters of auxin biosynthesis and signalling genes and likely PIF7 and PIF4 are dependent on each other during the induction of thermomorphogenesis by forming heterodimers [38]. Downstream of these signalling events, diverse chromatin remodelling, hormone-mediated signalling events and transcriptional processes have a role in translating the temperature information into proper responses to the prevailing temperature (reviewed in [2, 19, 20].

### Cold acclimation

Acclimation to low, but non-freezing temperatures, is called cold acclimation. In *Arabidopsis thaliana*, cold acclimation occurs at temperatures between 0 and 5 °C and leads to major transcriptional changes to induce physiological and biochemical modifications, resulting in enhanced freezing tolerance [39, 40]. Low temperatures amongst others triggers, growth inhibition, changes in cell wall composition to maintain cell wall integrity, capture of Reactive Oxygen Species (ROS), production of cryoprotective proteins, increased osmolyte levels and adjustments in photosynthesis [41–45]. Key to cold acclimation is the ICE1-CBF-COR regulon [39, 38, 44]. In response to cold the basic-helix-loop-helix type transcription factor INDUCER OF CBF EXPRESSION 1 (ICE1) binds to the promoter of *C-REPEAT BINDING FACTOR* (*CBF*) genes. In turn, the *CBF* genes (*CBF1*,* CBF2* and *CBF3*) bind to the *cis*-element of the *COLD RESPONSIVE* (*COR*) genes, thereby activating their expression. This leads to the induction of cold acclimation responses, including induction of cryoprotective proteins that protect plant cells against cold stress-induced damage to the membrane [39]. ICE1 is mostly regulated at the post-translational level by protein kinases, such as MITOGEN ACTIVATED PROTEIN KINASES (MAPKs), that can either stabilize or de-stabilize the ICE1 protein by protein phosphorylation (reviewed in [4]).

### Studying temperature acclimation across the temperature spectrum

The molecular regulation of thermomorphogenesis and cold acclimation is relatively well understood. To the best of our knowledge however, none of the identified molecular factors have an apparent role in acclimation to both cold and warmth, despite being part of the same temperature continuum. Investigating and identifying molecular factors that regulate acclimation processes along the temperature spectrum are considered prime targets for the development of climate tolerant crops [7]. It thus is essential to study plant acclimation responses across a gradient of applied temperatures (i.e. perform temperature dose-response assays). This is not only true for responses to abiotic stresses, but also for biotic stresses, as both cold and warm ambient temperatures also have an effect on plant immune responsiveness [47, 48].

Studies that cover abiotic signal gradients are very common in ecological research [49–53]. Although studying spatial and temporal gradients that occur naturally can explain variation on a large scale, such studies also typically include many confounding factors such as fluctuations in precipitation, soil type and differences in, for example, wind and light exposure. Therefore, if one’s aim is to understand the effect triggered by a change in a single environmental factor, geographical gradients, such as elevation and latitudinal gradients, are often relatively poor proxies [54]. This might be especially true when one wants to associate mild changes in temperature to growth and development, as the natural stochasticity in the temperature signal (momentaneous) and rhythmicity (diurnal and seasonal changes) both may hamper detection of causal relations between mild/small non-stochastic changes, such as consistent average climate warming, in temperature values and plant trait effect sizes. This is especially true in non- or semi-controlled field environments, where light quality and quantity and other parameters can strongly fluctuate. In the context of temperature studies, consistency in light intensity and quality is crucial, as major parts of the temperature and light signalling networks overlap and have the potential to evoke very similar responses such as, e.g. elongation of the hypocotyls and petioles [55–59]. Given these confounding effects, studying effects of temperature dose on plant traits thus ideally requires a stable research environment (laboratory climate-controlled rooms, cabinets or greenhouses) where temperature is the only parameter that is empirically tweaked. Studying plant temperature dose-responses over a wide temperature gradient is however a technical challenge; i.e. each temperature setting would require one cabinet or growth room. To overcome these limitations we considered whether thermal gradient tables, that are commercially available and often used for e.g. assessing seed quality by breeders [60, 61], are suitable for studying plant traits over a temperature gradient within the same confined experimental set-up under otherwise controlled environmental conditions (e.g. fixed photoperiod, humidity, light quantity and quality). We describe thermodynamic and technical aspects of our thermal gradient table setup and validate the table by demonstrating that diverse typical morphological, (molecular) physiological and developmental aspects of cold acclimation and thermomorphogenesis can be recapitulated by using our set-up; including temperature dose-response effects on seed germination, hypocotyl elongation, leaf development, hyponasty, temperature marker gene expression, ion leakage, hydrogen peroxide levels, stomatal conductance, chlorophyll content and rosette growth. We provide detailed technical information, growth protocols and considerations to aid the research community in rapid incorporation of thermal gradient table systems into the research field of temperature signalling and response.

## Materials and methods

### Thermal gradient table

The custom-made thermal gradient table used in this study was developed and constructed by Flohr Instruments (Nieuwegein, the Netherlands) in consultation with the authors. The table’s dimensions are 1135 mm by 1745 mm and is 1170 mm high. The table uses standard voltage (230 V/ 50 Hz) and has a power of 1.5 kW. The thermal gradient table houses an aluminium plate that is heated or cooled from either side by water (water pressure 1–5 bar) creating a temperature gradient. The maximum set temperature difference that can be created in one experiment is approximately 20 °C with a minimum set temperature of 5 °C and a maximum of 40 °C. The table is equipped with specialized overhead-placed Valoya BX120c3 LightDNA NS1/FR LED lighting, including optional dimmable far-red light and is placed in an air-conditioned dark room set at 21^o^C. Reported temperatures throughout this study refer to the set (table) temperatures.

### Plant material and growth conditions

*Arabidopsis thaliana* Col-0 seeds were obtained from the Nottingham Arabidopsis stock centre (http://arabidopsis.info/). The *pHSP70::LUC* line was kindly gifted by Phil Wigge [31]. *pPIF4::PIF4-LUC* was kindly gifted by Sander van der Krol [62]. *pICE1::LUC* was generated in our lab using Golden Gate cloning (method described below).

**Fig. 1:**
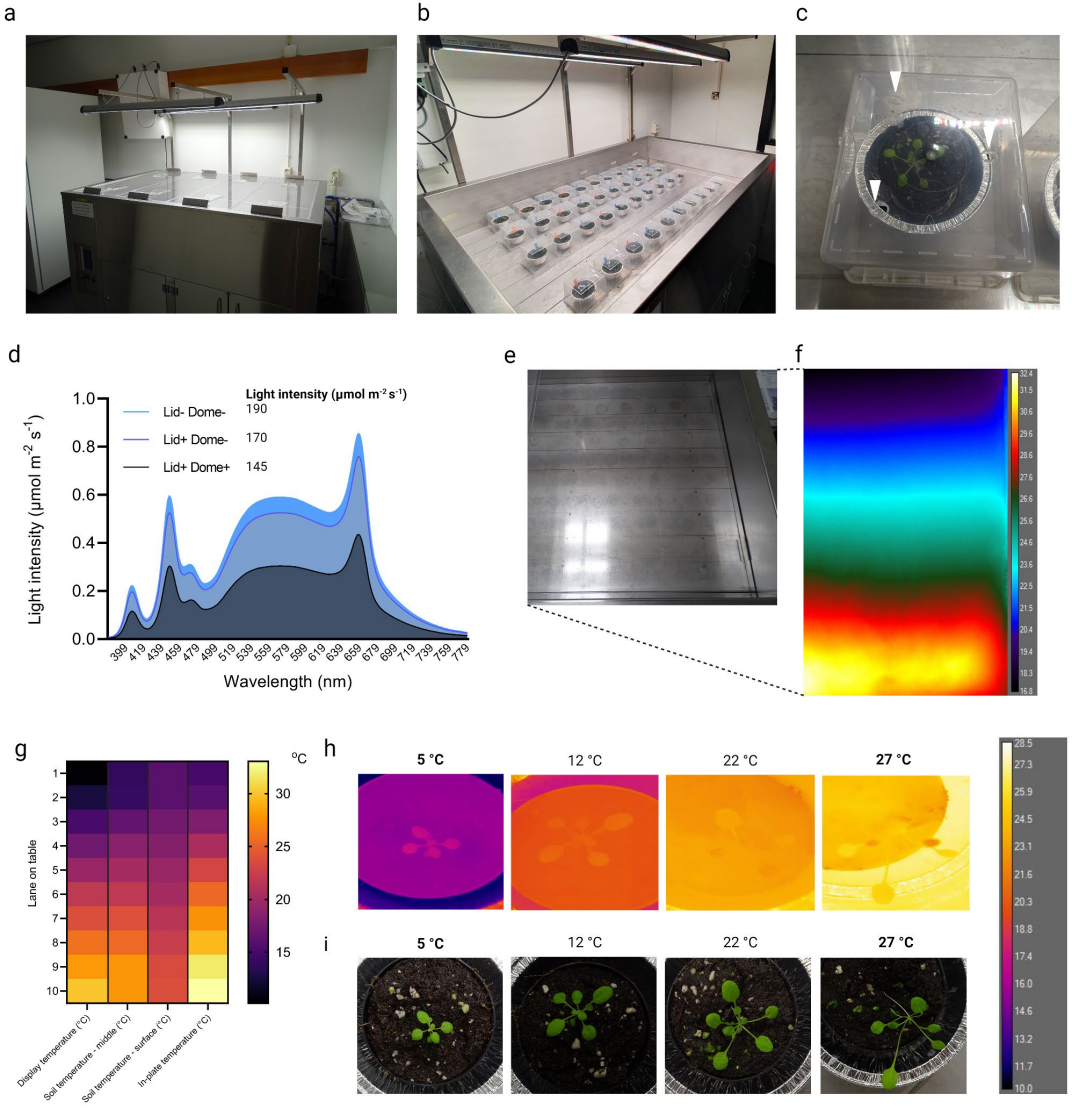
Thermal gradient table specifications and settings. (**A**) Exterior of the thermal gradient table used in this study. Note the LED lights mounted above the table and the removable Plexiglas lids that cover the growth space. (**B**) Typical temperature dose-response experiment using *Arabidopsis thaliana*. Plants are grown in plastic pots on soil and placed in aluminium muffin cups and covered by plastic domes. Note that each indicated row along the long axis of the table is a temperature treatment and that during the experiments the table was closed with the lids that are visible in (**A**). (**C**) Top picture of an individual *Arabidopsis thaliana* plant grown in plastic pot, inserted in the aluminium muffin cup. A dome containing three holes (indicated by white arrows) was placed over the cup. (**D**) Light spectrum (graph) and intensity (µmol m^− 2^ s^− 1^, noted next to legend) with or without dome and Plexiglas lids covering the table. (**E**) Top view of the thermal gradient table on which the ten treatment lanes are visible. (**F**) Pseudo-coloured infrared (thermal) image of part of the thermal gradient table depicted in (**E**) showing the temperature range with low temperature at the top of the picture and high temperatures on the bottom of the picture. On the right of the picture the colour legend can be seen. Here the gradient was from 10 °C to 32 °C. (**G**) Heat map showing the temperature for each lane on the table (row 1 to 10). Indicated are the display (i.e. set) temperature (left column), actual soil temperature measured in the middle of the pot (middle-left column), surface temperature of the soil (middle-right column) and in-plate temperatures of MS-agar-containing petri dishes placed horizontally on the table (right column). The gradient set on the table was 10 °C to 32 °C. (**H**) Infrared thermal images of three-week-old Col-0 wild type plants grown on a gradient set from 5 °C to 27 °C. Set values are highlighted in bold. A colour legend for the false-coloured infra-red image is included on the right. (**I**) RGB images of the plants used for Infra-Red imaging in (**H**)

Tomato (Moneymaker, LOT.C.20171-3) and lettuce (butterhead, variety “Larissa”, LOT.2014-3) were commercially obtained from www.moestuinland.nl, brand “Sluis Garden”. Unless stated otherwise, seeds were sown and grown in plastic round pots (Brinkman Agro, 4 cm diameter) containing a mix of potting soil and perlite (Primasta BV, Asten, The Netherlands) and subsequently stratified at 4 °C in darkness for four days. The pots were then shifted to the thermal gradient table set at 21 °C for 7 days. Thereafter, seedlings were transplanted to individual plastic round pots (Brinkman Agro, 4 cm diameter) on potting soil and perlite (Primasta BV, Asten, The Netherlands), inserted into aluminium muffin cups, and covered by plastic tissue culture boxes (Duchefa Sterivent high container 107 × 94 × 96 mm) with three added holes (2 × 7 mm) for aeration that were placed upside down over the plants that were allowed to acclimate for 2 days after transplantation at 21 °C. Thereafter, the thermal gradient table was set to the indicated gradients and plants were left to grow until phenotypic analysis. The lights above the thermal gradient table were set to short-day photoperiod (8-h light/16-h darkness), 100 to 150 µmol m^− 2^ s^− 1^ photosynthetic active radiation (PAR) white-light conditions (for spectrum and light intensity see Fig. 1D). Light spectrum and light intensity were measured using a LI-COR LI-180 spectrometer. For plate experiments square sterile petri dishes (Greiner Bio-One, 688,161) were used. Unless stated otherwise, the plates contained 50 ml 0.8% plant agar, full strength Murashige-Skoog (MS including MES Buffer and vitamins, Duchefa) medium without sucrose, that were closed with ventilating Micropore tape (3 M) after sowing. The seeds were stratified for four days at 4 °C before transferring to the thermal gradient. Plates were placed horizontally on the table. In the higher temperature range limited condensation occurred. Excess water was removed once a week with a syringe and sterile needle.

### Generation of transgenic *pICE1::LUC* line

To generate the transgenic *pICE1::LUC* line, the promoter (here determined as 1500 base pairs upstream of the start codon of the *ICE1* (*AT3G26744*) coding sequence) was synthesized using Integrated DNA technologies (IDT) Gene Synthesis services. The original sequence (www.arabidopsis.org) has been altered to make it compatible for Golden Gate cloning. These changes included adding overhangs and *Bpil* restriction sites at the start and end of the sequence, as well as removing internal restriction sites (Supplemental table S1). For the *LUCIFERASE* coding sequence a standard plasmid from the MoClo Plant Parts Kit [63] (Addgene), was used (*pICSL80001*). In addition, the phosphinothricin resistance cassette [63] was used (*pICSL70005*, Addgene). Used primers, vectors and plasmids are indicated in Supplemental table S2 and S3 respectively. The construct was developed using the Golden Gate cloning technique, making use of the T4 DNA ligase and the restriction enzymes *Bpil* and *BsaI* (Thermo Scientific) [61, 62]. The level 0 constructs were put into specific entry vectors (Supplemental table S2). Thereafter, the created *pICE1::LUC* level 0 construct was put into the level 1 position entry vector (*pICH47742*). The level 0 Basta resistance cassette (*pICSL70005*) was put into the level 1 position 1 entry vector (*pICH47732*) and was added to all other level 1 constructs separately into a level 2 entry vector (*pAGM4673*) making use of an end linker 2 (*pICH41744*). Every construct was transformed into heat-shock competent *Escherichia coli DH5α* cells at 42 °C, selected for by blue/white screening, purified using the E.Z.N.A. Plasmid Mini Kit I (Omega Biotek) and confirmed by sequencing the whole fragment. Level 2 constructs were transformed into electrocompetent *A. tumefaciens* strain *C58* and transformed into Col-0 plants using the floral dipping method [64].

### Quantitative real-time PCR (qRT-PCR)

10 day-old Arabidopsis seedlings were harvested at dawn (start of the photoperiod) and flash-frozen in liquid nitrogen before storage at -80 °C. Each sample for qRT-PCR contained more than 20 seedlings. The experiments included three to four biological replicates (each of > 20 seedlings) and two technical replicates. RNA was isolated as described previously [65]. The qRT-PCR reactions were performed using SYBR green mastermix (Life Technologies) on a ViiA7 Real Time PCR system and ViiA7 software was used to analyse the data. Relative expression levels were calculated using the ΔΔCt method [66] and normalized to the expression of the reference gene: *AT4G05320*. See supplemental table S2 for qRT-PCR primers used in this study.

### Quantitative luciferase assay

Col-0, *pHSP70::LUC* [31], *pICE1::LUC* and *pPIF4:PIF4-LUC* [62] seeds were gas-sterilized by chlorine gas for 3 h. The sterile seeds were sown on sterile Petri dishes (Greiner Bio-One, 688,161) containing 0.8% plant agar, full strength Murashige-Skoog medium as indicated above, without sucrose. After stratification, the plates were placed horizontally on the thermal gradient table set at 21 °C under short day conditions (8-h light/16-h darkness) and pre-germinated for 3 days. After 3 days the table was set to a gradient ranging from 5 °C to 27 °C. The seedlings were harvested on day 10 at dawn (start of the photoperiod) and immediately snap frozen in liquid nitrogen before storage at -80 °C.

Quantitative LUCIFERASE (LUC) assays were performed as described in [65]. Protein extracts were prepared from ~ 30 10 day-old seedlings that were snap-frozen in liquid nitrogen. The plant material was ground to fine powder using 3 mm glass beads and a TissueLyser II (Qiagen), after which 100 µl 1x passive lysis buffer (PLB, Promega E1941) was added. The mixture was homogenized by vortexing, followed by 10 min incubation at room temperature. Debris was pelleted by centrifugation and 20 µl of supernatant was transferred to a 96-well Lumitrac plate (Greiner Bio-One). The LUC activity was determined using the LUC Assay System detection kit (Promega, #E1500) in a Glomax 96 microplate luminometer (Promega, #E6521), with the “LUC Assay System with Injector” protocol (2-s delay between injection and measurement, 10-s integration time). The protein concentrations were determined for each sample by adding 200 µl Bradford Reagent (Sigma-Aldrich, #B6916) to 4 µl sample supernatant in a clear 96-well plate. Absorbance was measured using a Biotech synergy HT-plate reader. A Bovine Serum Albumin (BSA) (Sigma-Aldrich, #A7906) standard curve was included to calculate protein concentrations, to normalize the LUC signal to the protein concentration of each sample. In each replicate Col-0 wild type seedlings were included as negative control. The experiment was repeated five times.

### Phenotyping

#### Germination assays

Fully after-ripened *Arabidopsis thaliana* Col-0 wild type seeds were sown on wet filter paper and placed in small round petri-dishes (Greiner Bio-One, 628,161). After sowing, the seeds were stratified for four days at 4 °C in darkness to synchronize the ability to germinate. Thereafter, the petri-dishes were placed horizontally on the thermal gradient table and placed at different set temperatures ranging from 12 °C to 32 °C. Germination was counted every 24 h, shortly after the start of the photoperiod, for four days. We considered a seed germinated when the radicle penetrated the seed coat. Every petri-dish contained 50 to 130 Arabidopsis seeds. The experiment was repeated four times per temperature condition.

#### Hypocotyl measurement

Arabidopsis seedlings were cultivated from sterilized seeds on sterile 0.8% agar, full strength Murashige-Skoog (MS including MES Buffer and vitamins, Duchefa) medium without sucrose on square sterile petri-dishes (Greiner Bio-One, 688,161) as indicated above, unless stated otherwise. The seeds were stratified for 4 days at 4 °C in the dark to synchronize germination. Thereafter, the plates were placed horizontally on the thermal gradient table and grown for 48 h at 21 °C. The plates were then subjected to the different treatment temperatures and seedlings were left to grow until they were 10 days old. The plates were then scanned using a flatbed scanner and hypocotyl lengths were measured using ImageJ image-analysis software (https://imagej.nih.gov/ij/).

Lettuce seeds were sown in tissue culture boxes on wetted filter paper and grown on the thermal gradient table on a gradient set from 5 °C to 27 °C in long day conditions (16-h light/ 8-h darkness). Hypocotyls of 20 to 30 seedlings per temperature were measured when the seedlings developed the first true leaves using ImageJ.

#### Rosette trait phenotyping

Arabidopsis plants were grown as described above, on pots containing potting soil and perlite (Primasta BV, Asten, The Netherlands), until they reached 10 true leaves. Each 5th youngest leaf was labelled and the leaf was then imaged from the side with a standard digital camera. Petiole angle was measured between the petiole/lamina junction and a fixed basal point of the petiole (rosette base), relative to the horizontal (see Fig. 4C). Thereafter, the plants were cut at the root/shoot junction and the rosette was pressed flat to take a picture from the top. Petiole length was measured between the base of the leaf and the central point of the rosette (see Fig. 4C). Both petiole angle and length were measured using ImageJ image-analysis software (https://imagej.nih.gov/ij/).

Lettuce plants were grown on potting soil and perlite (Primasta BV, Asten, The Netherlands) in long day conditions (16-h light/8-h dark) on a gradient set from 5 °C to 27 °C until their reached 6 true leaves. Leaf and petiole length (mm) were measured on leaf number 3 to 5 and then averaged.

For time course analysis of hyponasty, Col-0 plants were cultivated on potting soil and perlite (Primasta BV, Asten, The Netherlands) in short day conditions (8-h light/16-h darkness) at 21 ^o^C until they reached 10 leaves. Plants were then separated in two treatment groups and each group was moved to either end of the thermal gradient table set at 21 ^o^C short days conditions the day before the experiment started. Leaves that were obscuring the petiole base from the perspective of the middle of the table were removed. At the onset of the next day, the table was set to a gradient of 21 ^o^C (perceived by the control group) to 32 ^o^C (perceived by high temperature treatment group). Subsequently, plants were manually photographed each hour throughout the photoperiod. Petiole angle was measured between the petiole/lamina junction and a fixed basal point of the petiole (rosette base), relative to the horizontal using ImageJ (http://rsb.info.nih.gov/ij). Of each plants, two petioles were measured and averaged per timepoint. Soil temperature was measured every 10 min in the first hour after starting the experiment and every hour thereafter using a digital thermometer (Prima Long, Amarell Electronic) that was inserted halfway into the pot of soil.

### Physiological assessment

#### Chlorophyll content and stomatal conductance

*Arabidopsis thaliana* chlorophyll content and stomatal conductance were measured of the 5th youngest leaf when plants reached the stage of 10 true leaves. Chlorophyll content was measured using a Chlorophyll Conductance Meter (Eijkelkamp CCM-300). Three measurements of the same leaf were taken and averaged. Stomatal conductance was measured using a Leaf Porometer (METER SC-1, Decagon Devices, Inc., Pullman, USA) on the same leaf.

For lettuce chlorophyll content, plants were grown in long day conditions (16-h light/8-h darkness) on a gradient set from 5 °C to 27 °C. Chlorophyll content was measured of plants with 6 true leaves. The measurement was taken on the second youngest leaf. Three measurements of the same leaf were taken and averaged.

#### Ion leakage assay

Col-0 wild type plants were grown on soil as described above on a gradient set from 5 °C to 27 °C in short day conditions (8-h light/16-h dark). Plants were grown in each temperature condition until they were 3 weeks old. To determine ion leakage, the plants were cut at the base of the rosette. The whole rosettes were rinsed in deionized water and transferred to a 15 ml Griner tube (CELLSTAR, Greiner Bio-One) containing 10 ml deionized water. The tubes were shaken at room temperature for 1 h. Thereafter, 100 µl of solution was pipetted onto an Horiba LAQUAtwin-EC-33 conductivity meter to determine the initial conductivity. After measuring, the remaining solution was incubated in a water bath at 95 °C for 30 min. After cooling down to room temperature, 100 µl of the solution was pipetted onto the conductivity meter to determine the final conductivity. Ion leakage described above on a set gradient fromwas calculated as: Initial Conductivity / Final Conductivity * 100%. For each temperature conditions six biological replicates were included.

#### DAB staining

Col-0 wild type plants were grown on soil as described above on a set gradient from 5 °C to 27 °C in short day conditions (8-h light/ 16-h darkness). Plants were grown in each temperature condition until they were 3 weeks old. The plants were cut at the base of the rosette and placed into 6 well multi-well plates (Greiner Bio-One) and submerged in 1 mg/ml 3,3’-diaminobenzidine (DAB) solution, in 1x phosphate-buffer saline (PBS). The plates were covered by aluminium foil and placed in a vacuum desiccator for 15 min in darkness. After being exposed to a vacuum, the DAB solution was replaced by fresh DAB solution and again placed in a vacuum for 15 min. Thereafter, the plates were transferred to a shaking table and left shaking overnight at room temperature. The next day the DAB solution was removed and replaced by de-staining solution (acetic acid, glycerol, 96% ethanol, 1:1:3) and placed in an oven at 60 °C. After 60 min, the whole rosettes were transferred to a new plate containing 6 ml 90% lactic acid. Thereafter, the plates were scanned using a flatbed scanner. Acquired thermal images were analysed using ImageJ software (https://imagej.nih.gov/ij/). For each temperature six biological replicates were included.

### Temperature measurements

To measure soil surface temperature, to visualize the surface temperature of the thermal gradient table and plant leaf temperature, a FLIR A655sc High Resolution LWIR thermal imaging (IR) camera was vertically mounted above the thermal gradient table, equipped with a 13.1 mm FoV 45°x 33.7°hawkeye IR lens and connected to a laptop. A thermal image was taken using FLIR ResearchIR Max 4 software. Acquired thermal images were analysed using ImageJ software (https://imagej.nih.gov/ij/). For imaging the thermal gradient table, surface wet paper was placed on top of the table to account for the reflective properties of the stainless-steel casing and aluminium plate. The Plexiglass lids of the table were removed before imaging.

To measure plant leaf temperature Col-0 plants were grown on the thermal gradient table set from 5 °C to 27 °C until they were three weeks old. For imaging, a single plant was placed in an aluminium case (with a hole at the top for the thermal imaging camera, see Fig. S1E) on the table lane on which the plant has been growing. The thermal imaging camera was vertically mounted in the case hole and was left to image the plant for an hour, taking an image every 10 min. This process was repeated for each lane. Acquired thermal images were analysed using ImageJ software (https://imagej.nih.gov/ij/). For each plant the leaf temperature of leaf number 3, 4 and 5 were measured.

Soil temperature was measured using a digital thermometer (Prima Long, Amarell Electronic) that was inserted halfway into the pot.

### Statistical analysis and data visualisation

Data visualization and statistical analysis was performed using GraphPad Prism 10 (version 10.1.1323, GraphPad Soqware, La Jolla, USA). Depending on the data, a one-way or two-way ANOVA with Tukey’s *post hoc* test was performed. Figures were created with www.BioRender.com.

## Results

### Thermal gradient tables can be used for temperature dose-response assays in plants

We tested if a thermal gradient table can be used to study temperature dose-dependent responses in plants within a single and confided fully-controlled experimental setup (Fig. 1A, B). The stainless-steel thermal gradient Table (1745 × 1135 × 1170 mm) used in this study consists of an aluminium plate that can be heated or cooled at the side (long axis) of the table. The table can be closed with 3 independent Plexiglass lids, with the option to control aeration via manually adjustable sliders covering holes in the lids (supplemental Fig. S1A). To contain the temperature emitted from the aluminium plate and to retain high humidity for plant growth, plastic domes were placed over the growing plants (Fig. 1B, C). Additionally, the placement of the domes helps to limit temperature-dependent soil water loss. In order to enhance temperature conductivity from table to soil, the plastic pots containing a single plant were placed in aluminium cups (Fig. 1C). The table is equipped with intensity-adjustable LED lighting with the option of adding extra far-red light, that provides consistent light quality with and without the use of the Plexiglass lids and plastic domes (Fig. 1D, Fig. S1B).

The temperature of the aluminium plate is monitored by 10 sensors divided over the short axis of the table, thereby dividing the table in 10 lanes that each differ in temperature (Fig. 1E). The average temperature of each row is dependent on the proportional difference between the set temperatures of the outer rows (outer sides of the table), as visualised using infra-red thermal imaging (Fig. 1F). The temperature of the first and last row can be precisely set via a touchscreen display (Fig. S1C) and the temperature of each of the rows in-between is monitored and displayed (Fig. S1D).

To determine whether the set (two outer rows) and monitored (eight rows in-between) temperatures translate to actual soil temperature, soil surface temperature, belowground temperature and in-plate temperature was measured (Fig. 1G). As the pots are heated or cooled from below, the soil surface temperature expectedly deviated slightly from the read-out and set temperatures. Nonetheless, when the table was set to generate a gradient from 10 °C to 32 °C the below ground temperatures ranged from 13 °C to 28 °C, respectively and from 16 °C to 25 °C on the surface (Fig. 1G). To check if the temperature of the table translates to differences in leaf temperature, *Arabidopsis thaliana* Col-0 wild type plants were grown on a gradient set from 5 °C to 27 °C for three weeks. Leaf temperature measurement by infra-red thermal imaging showed that both soil and leaf temperature indeed scale with the set table temperature settings (Fig. 1H). Of note, leaf and soil temperature were slightly higher in the lower end of the gradient. While on the higher end of the gradient, leaf and soil temperature were slightly lower than the set temperature (Fig. S1E, F), in consistency with the soil surface temperature data shown in Fig. 1G. Figure 1H also shows the compact appearance of cold-grown plants and the typical ‘open rosette’ architecture of plants grown at warm temperatures.

### Seed germination is inhibited at high temperatures and germination is delayed at low tempplate is monitored by 10 sensors divided over theeratures

We next tested if applied temperature gradients could recapitulate known morphological, molecular, physiological and developmental aspects of plant growth from seed to mature plant.

From literature it is known that temperature treatments affect the total number of germinating seeds and the onset of germination of viable seed batches. For instance, in cucumber (*Cucumis sativus*), mung bean (*Phaseolus aureus*) and white mustard (*Sinapis alba*), temperatures below 11 °C severely impact a seed’s ability to germinate [67]. In *Arabidopsis thaliana* both ends of the physiological temperature spectrum negatively impact germination, albeit differently. Temperatures above 32 °C negatively impact the number of seeds that germinate [66–68], whereas low temperatures mostly delays the moment of germination, without affecting the fraction of seeds that eventually germinate. Even at 4 °C up to 80% of Arabidopsis wild type seeds can germinate, but this can take up to 264 h [69].

**Fig. 2:**
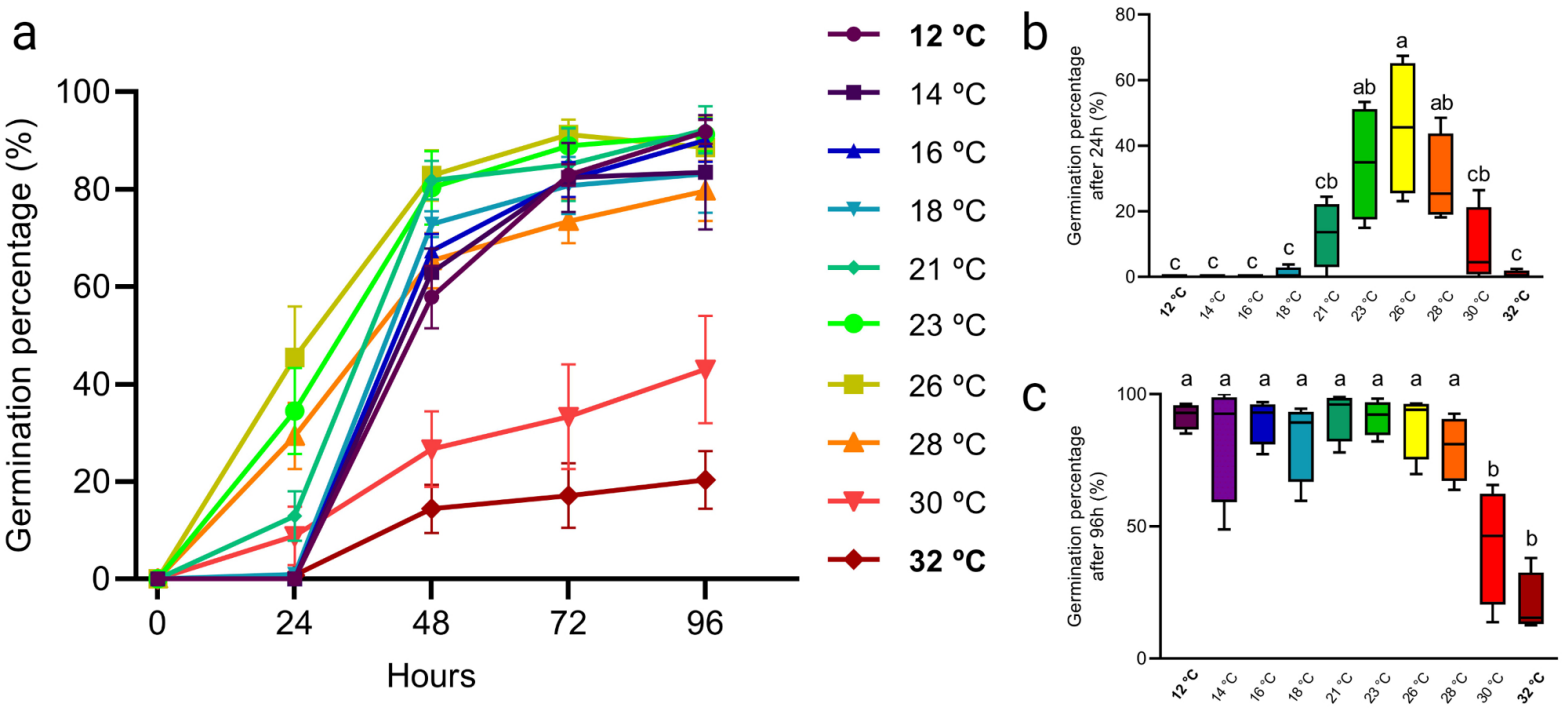
Temperature-dose effect on germination of *Arabidopsis thaliana* Col-0 wild type seeds. (**A**) Fraction of seeds germination over time. For each applied temperature, 50–130 Arabidopsis Col-0 wild type seeds were sown on wet filter paper in petri-dishes that were placed at different temperature conditions (rows) on the thermal gradient table. Germination was scored every 24 h for four days (96-h). The experiment was repeated four times. Error bars represent standard error of the mean. (**B**,** C**) Germination percentage at different temperatures at 24 (**B**) and 96 (**C**) hours respectively. Letters indicate statically significant differences (*P < 0.05*) as determined by one-way ANOVA with Tukey’s *post hoc* test. The table was set to a gradient ranging from 12 °C to 32 °C. These set values are highlighted in the graphs in bold in the legend (**A**) or on the x-axis (**B**, **C**). The temperatures in-between these set temperatures are an average of the displayed setting on the table for each specific lane

To test whether these temperature effects on seed germination could be recapitulated on our thermal gradient table we exposed viable *Arabidopsis thaliana* Col-0 wild type seeds to a set gradient of 12 °C to 32 °C. The fraction of germinated seeds was scored every 24 h until day 4 (96 h; Fig. 2A). Ambient high temperatures seem to be optimal for seeds germination, as at 26 °C approximately 45% of seeds germinated after 24 h (Fig. 2B). As expected, low temperatures led to lower levels of seed germination (Fig. 2B). Approximately 80–90% of seeds germinated after 96 h when exposed to 12 °C to 28 °C, whereas temperatures of 30 °C and above leads to a significant reduction of the fraction of germinating seeds (Fig. 2C). This data suggest that in agreement with literature seed germination is significantly negatively affected by temperatures over 30 °C (Fig. 2B) and low temperatures merely slow down germination speed. We therefore conclude that our thermal gradient table is suitable for assessing seed germination, and accordingly likely other seed traits such as dormancy levels, across the temperature spectrum.

### Hypocotyl elongation scales with applied temperatures

**Fig. 3:**
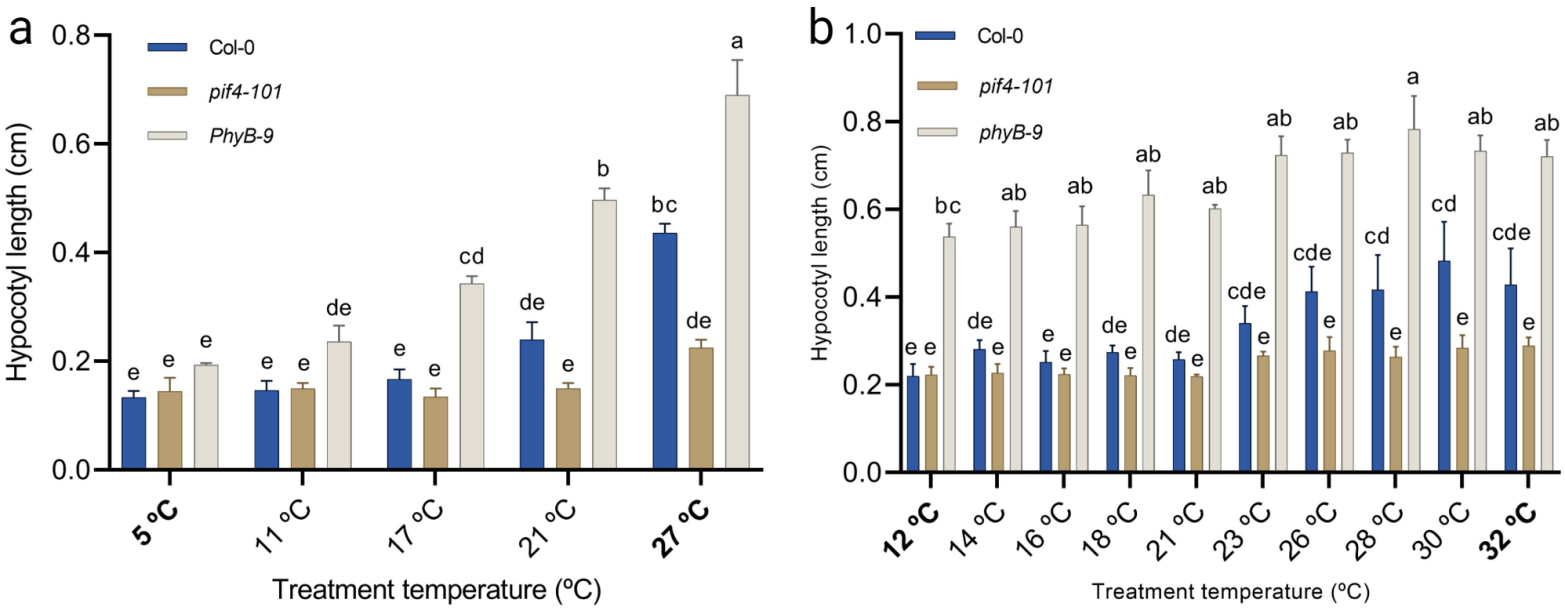
Temperature-dose effect on hypocotyl elongation. (**A**, **B**) Col-0, *pif4-101* and *phyB-9* seeds were sown on plates containing 1x MS 0.8% agar and grown in short day conditions (8-h light/16-h darkness). Hypocotyls of approximately 30 10 day-old seedlings were measured per temperature condition. The experiment was repeated three times. Letters indicate statically significant differences (*P < 0.05*) as determined by two-way ANOVA with Tukey’s *post hoc* test. Error bars represent the standard error of the mean. The table was set to a gradient ranging of (**A**) 5 °C to 27 °C and (**B**) 12 °C to 32 °C (indicated in bold). The temperatures in-between are an average of the displayed setting on the table for each specific lane

Seedling hypocotyl elongation is one of the earliest signs of thermomorphogenesis and can be observed in many different *Arabidopsis* accessions, as well as in crop species such as cabbage *(Brassica oleracea*) and tomato (*Solanum lycopersicum*) [2, 19, 20]. Hypocotyl elongation is proposed to move the sensitive meristematic and photosynthetically active tissues away from warm soil and is considered to promote plant cooling by allowing air movement around the shoot [21, 24]. Previous research that assessed hypocotyl length of seedlings grown from 16 to 28 °C has shown that hypocotyl length positively correlates with temperature [22]. The study of Ibañez and colleagues also demonstrated that the genetically-determined extent of hypocotyl elongation at a given temperature is a good predictor of temperature sensitivity of other morphological and phenological traits that occur later in the life of the plant. To test whether our thermal gradient table setup is suitable for assessing seedling responses to temperature dose, we first assessed how set temperatures relay to the inside of MS-agar containing petri-dishes. When the table was set to a gradient from 10 °C to 32 °C the in-plate temperatures ranged from 15 °C to 33.1 °C, respectively (Fig. 1G). Next, hypocotyl length was measured of 10 day-old Arabidopsis seedlings grown across two different temperature gradients, from 5 °C to 27 °C (Fig. 3A) and from 12 °C to 32 °C (Fig. 3B), in separate experiments. Mutants of *Phytochrome B* (*PhyB*) and of *PHYTOCHROME INTERACTING FACTOR 4* (*PIF4*), were included as these mutants exhibit opposite phenotypic responses to temperature with respect to the Col-0 wild type. Hypocotyls of *phyB* mutants are significantly longer than those of the wild type in both control and elevated temperature [32, 69–71]. On the contrary, *pif4* mutants display suppressed elongation under warm temperature conditions [29, 35, 72]. Overall, the hypocotyl lengths obtained on the thermal gradient table are very similar to those from regular growth cabinets in our lab [65, 73].

As expected, hypocotyls of Col-0 wild type seedlings significantly elongated at high ambient temperatures from ~ 23 °C onwards (Fig. 3B), as compared to the lower temperature range. When grown over a gradient set from 5 °C to 27 °C, Col-0 seedlings showed clear elongated hypocotyls at 27 °C (Fig. 3A). In all lower temperatures the Col-0 seedlings had hypocotyl lengths of around 2 mm. This is similar to the hypocotyl length of *pif4-101* seedlings that as expected were unable to elongate across the applied temperature gradients (Fig. 3A, B). Oppositely, *phyB-9* seedlings were constitutively more elongated than the wild type, independent of temperature (Fig. 3A, B). Although *phyB-9* hypocotyls were significantly longer than Col-0 wild type at all applied temperatures, the hypocotyl length of *phyB-9* seedlings did respond to temperature. This is in agreement with literature indicating that it requires a higher order *phytochrome* mutant (*phyABCDE*) mutant to fully loose temperature-dependent hypocotyl elongation [32]. We conclude that our thermal gradient table is suitable for assessing seedling traits across the temperature spectrum.

### Leaf hyponasty and petiole length increase with temperature in a dose-dependent manner

**Fig. 4:**
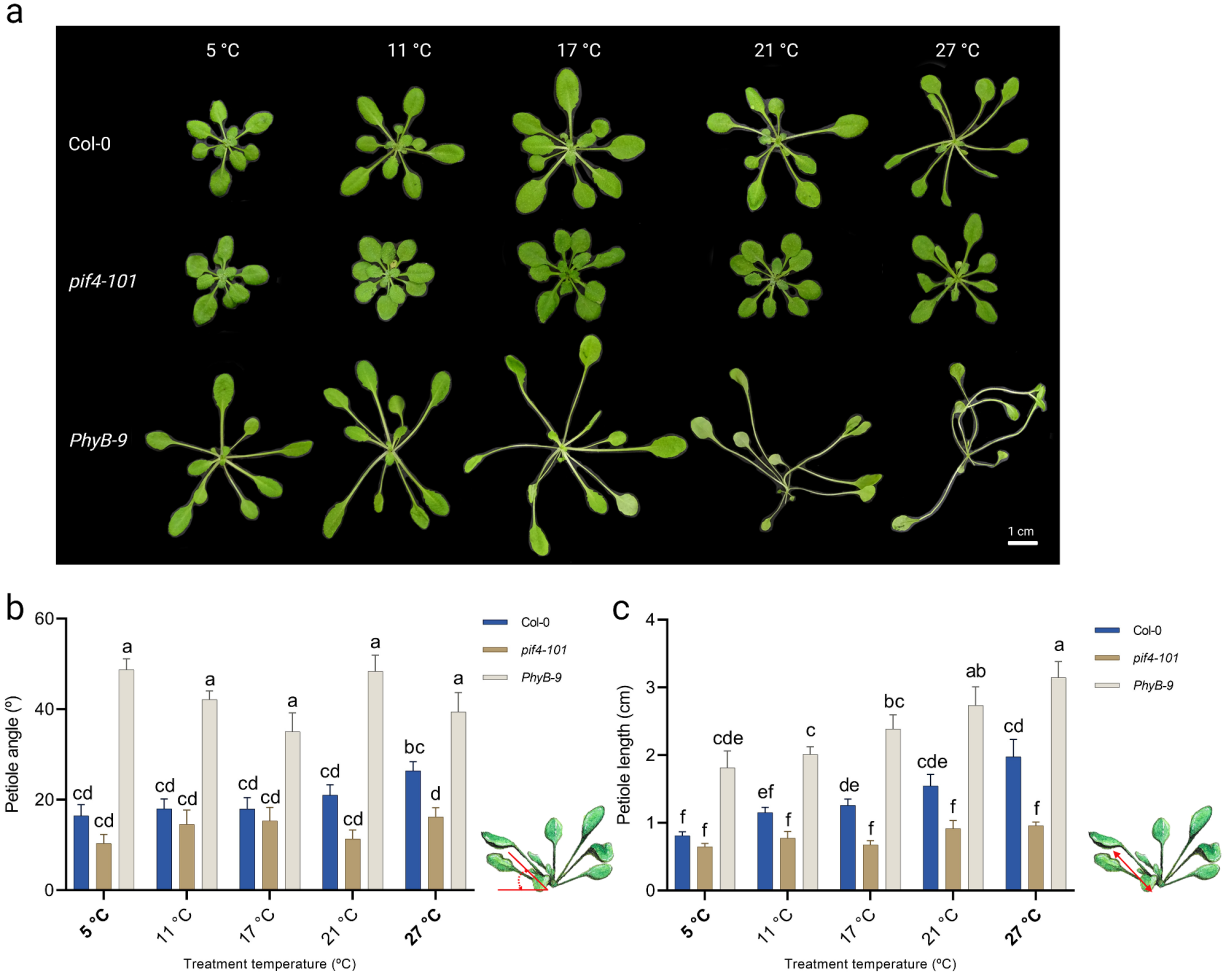
Temperature-dose effect on petiole length and leaf hyponasty. (**A**) Representative images of rosette-stage Arabidopsis Col-0, *pif4-101* and *PhyB-9* plants that were grown at different indicated temperatures on the thermal gradient table. Note the compact appearance of cold-grown Col-0 plants and the typical ‘open rosette’ architecture of Col-0 plants grown at warm temperatures. The table was set to a gradient ranging from 5 °C to 27 °C. Scale bar = 1 cm. (**B**) hyponasty (upward leaf movement) and (**C**) petiole lengths of the 5th youngest leaf of Col-0, *pif4-101* and *phyB-9* rosette plants at the 10 true leaf stage, grown on soil in short day conditions (8-h light/16-h darkness) on the thermal gradient table subjected to a gradient with set temperatures of 5 °C to 27 °C. The temperatures in-between are an average of the displayed setting on the table for each specific lane. Letters indicate statistically significant differences (*P < 0.05*) within a temperature treatment as determined by two-way ANOVA with Tukey’s *post hoc* test. Error bars represent the standard error of the mean. N = 9 plants for each condition. A schematic view of how petiole angle and petiole length were measured is provided in (**B**) and (**C**)

When growing Col-0 wild type rosette plants over a temperature gradient set from 5 °C to 27 °C on the thermal gradient table an ‘open rosette architecture’ (Figs. 1H and 4A) appeared at warmer temperatures. This appearance is typical for thermomorphogenesis and is considered beneficial for increasing the cooling capacity of the plant [19, 23, 24, 74]. On the other hand, at low temperatures plants typically stayed compact [41, 43] (Fig. 4A). As expected, *pif4-101* rosettes retain a compact appearance at every tested temperature. The *PhyB-9* mutant is constitutively elongated in all temperatures, yet increases in rosette size and petiole length with increasing temperature (Fig. 4A).

One of the component traits of thermomorphogenesis is elongation growth of the leaf petiole [2, 5, 19, 22]. Petiole elongation co-occurs with an increase in petiole- and leaf angle (hyponastic growth). The extent of hyponasty knowingly scales with temperature input [5, 22, 23, 75, 76] and initiates within 1 h after perceiving a high temperature cue. Petiole angles of Col-0 plants subjected to 32 °C started to deviate from those kept at control 21 °C between 1 and 2 h (Fig. S2A) whereas a soil temperature of 32 °C was reached ~ 1 h after switching the table from 21 °C to 32 °C (Fig. S2B). This suggests that plants sense – and respond to - the temperature cue conveyed by the thermal gradient table directly. After seven hours in 32 °C clear hyponastic movement of the leaves can be observed, creating a more open rosette in high temperature compared to 21 °C (Fig S2C-F). To quantify the extent of petiole elongation and hyponasty across the thermal gradient, Col-0 wild type, *pif4-101* and *phyB-9* mutant plants were subsequently grown over a gradient set from 5 °C to 27 °C until the rosettes had 10 true leaves. Petiole length and petiole angle were subsequently measured of the 5th youngest leaf (Fig. 4B). From 5 °C to 21 °C, Col-0 and *pif4-101* exhibit a consistent low petiole angle. Both Col-0 and *pif4-101* showed increased petiole angle at 27 °C, although Col-0 was, as expected, more responsive. In agreement with literature, the *phyb-9* mutant exhibited a constitutive enhanced hyponastic phenotype at all temperatures compared to Col-0 [64]. Similarly, petiole lengths showed a positive correlation with temperature (Fig. 4C) [22, 75, 77]. *Pif4-101* and *phyB-9* mutants showed the expected contrasting responses. Petioles of the *pif4-101* mutant did not elongate in response to temperature cues, whereas *phyB-9* mutants showed constitutively elongated petioles compared to Col-0 at all measured temperatures. Despite being constitutively long, *phyB-9* petioles elongated more at higher temperatures as compared to low temperatures (Fig. 4C). Both leaf hyponasty and petiole length thus increased in a temperature dose-dependent manner and we conclude from this that our thermal gradient table is suitable for assessing rosette traits across the temperature spectrum.

### Low ambient temperature negatively affects leaf initiation rate

**Fig. 5:**
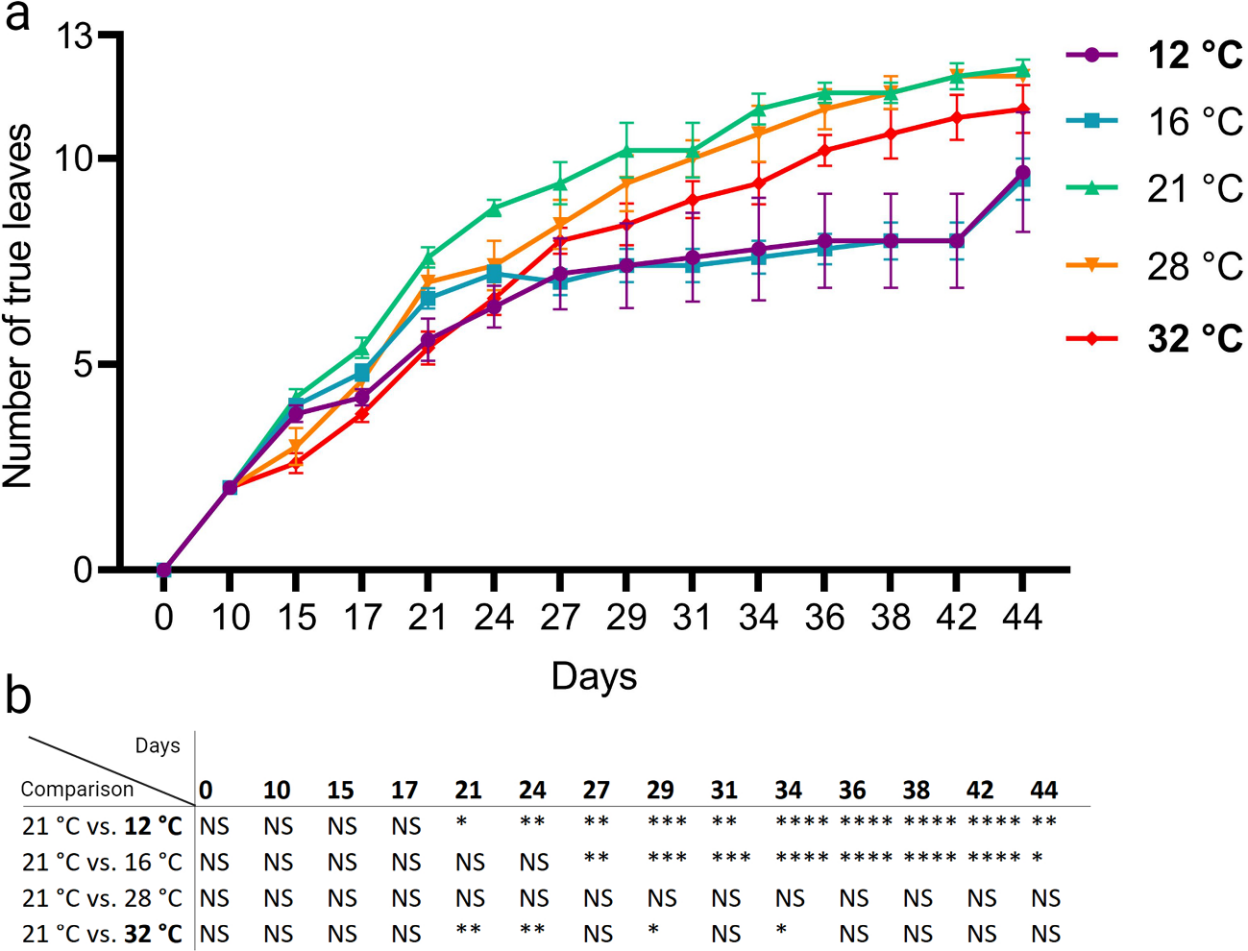
Temperature-dose effect on leaf initiation. (**A**) Col-0 plants were grown in soil in short day conditions (8-h light/16-h darkness) on the thermal gradient table with a gradient set from 12 °C to 32 °C (indicated in bold). The temperatures in-between are an average of the displayed setting on the table for each specific lane. Total number of true leaves (thus excluding cotyledons) per plant was counted until the plant reached 12 true leaves or until day 44. (**B**) Table showing statistical difference within each timepoint. Treatment temperatures (12 °C, 16 °C, 28 °C and 32 °C) are compared with 21 °C. Asterisk indicate statically significant differences (* P < 0.05, ** P < 0.01, *** P < 0.001, **** P < 0.0001) between different temperatures and 21 °C as determined by two-way ANOVA with Tukey’s *post hoc* test. NS = Not Significant. Error bars represent the standard error of the mean. For each temperature N = 5

All stages of leaf development are, at least to a certain extent, affected by temperature [59]. Previous research has shown that the rate of formation of new leaves (leaf initiation rate) in Arabidopsis is a carefully timed process that is directly related to temperature input. Leaf initiation rate is relatively fast at high temperatures (26 °C) and relatively slow at low temperatures (14 °C), compared to control temperatures (21 °C) [78]. To assess leaf initiation rate on the thermal gradient table, Col-0 wild type plants were grown on a gradient set from 12 °C to 32 °C (Fig. 5A). The number of true leaves (thus excluding cotyledons) was counted regularly until the plants reached 12 true leaves, or until day 44. In agreement with literature, leaf initiation was significantly slower in plants grown at low temperatures (12 °C and 16 °C) compared to plants grown at 21 °C (Fig. 5A and B). However, plants grown at relatively high temperatures (28 °C and 32 °C) did speed-up new leaf formation. Leaf formation was slightly, but significantly, slower in plants grown at 32 °C compared to their counterparts grown at control temperatures (Fig. 5A and B), suggesting that 32 °C is sub-optimal for leaf formation. We conclude that the thermal gradient table is suitable for assessing plant development and growth traits across the temperature spectrum.

### Hydrogen peroxide levels increase at low temperatures and ion leakage scales with temperature dose

**Fig. 6:**
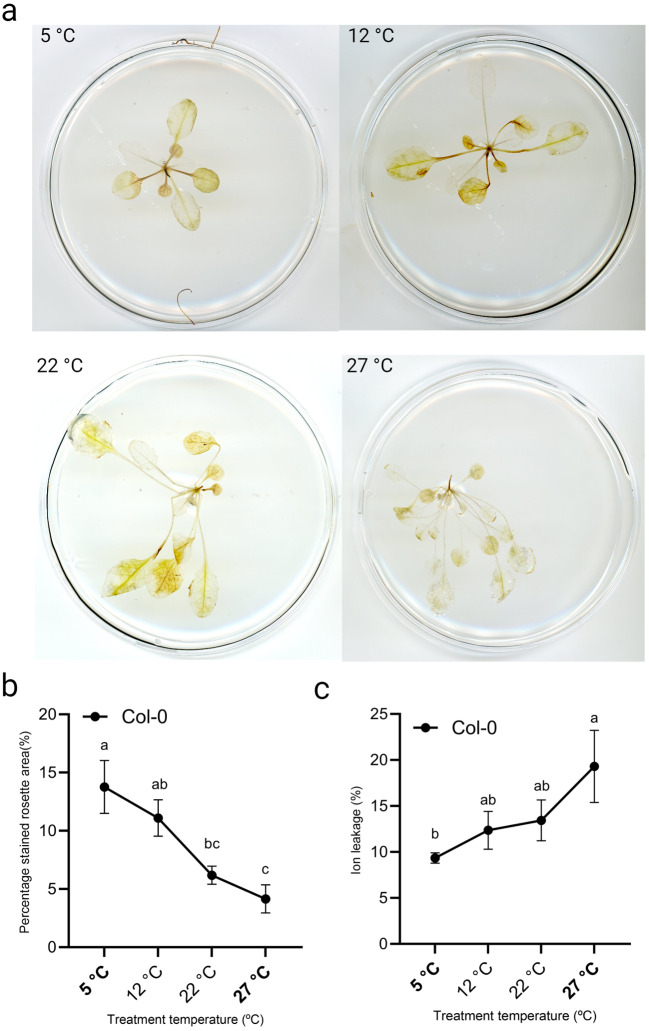
Temperature-dose effect on ion leakage and hydrogen peroxide levels (ROS). (**A**) Representative images of DAB-stained rosettes., (**B**,** C**) quantification of (**B**) DAB staining and (**C**) Ion leakage levels (%). Three week old Col-0 plants were used that were grown in soil in short day conditions (8-h light/16-h darkness) on the thermal gradient table with a gradient set from 5 °C to 27 °C (indicated in bold). The temperatures in-between are an average of the displayed setting on the table for each specific lane. Letters indicate statically significant differences (*P < 0.05*) within a temperature treatment as determined by one-way ANOVA with Tukey’s *post hoc* test. Error bars represent the standard error of the mean. N = 6 plants for each temperature condition

Previous work demonstrated that exposure to low temperatures leads to increased hydrogen peroxide levels [79], which could contribute to freezing tolerance [80]. To confirm this in our setup, Col-0 plants were grown for three weeks on a gradient set from 5 °C to 27 °C. Staining with 3,3’-diaminobenzidine (DAB) indicated a slight increase in hydrogen peroxide (H_2_O_2_) levels at low temperatures (5 °C and 12 °C) compared to control (22 °C) or high temperature (27 °C) (Fig. 6A). Quantification of stained rosette area confirmed a clear negative correlation with temperature, with significantly higher H_2_O_2_ levels at 5 °C (Fig. 6B).

Next, we assessed ion leakage levels, which is a proxy of cell death triggered by cold and heat stress [81] in Col-0 plants grown for three weeks on a gradient set from 5 °C to 27 °C. A positive, significant, correlation between ion leakage and temperature was found (Fig. 6C). Taken together, these results indicate that growing plants on our thermal gradient table affects plant responses to temperature on the physiological level, despite some other physiological parameters such as chlorophyll content and stomatal conductance did not clearly respond to temperature-dose in our setup (Fig. S3).

### High temperatures lead to increased *HSP70* transcription and PIF4 protein abundance in Arabidopsis seedlings, whereas low temperatures induce expression of cold-induced genes *KIN10* and *COR15A*

*HEAT SHOCK PROTEIN 70 (HSP70)* transcript levels are an output of the ambient temperature-sensing pathway and is considered a ‘molecular thermometer’ as *HSP70* mRNA levels scale with temperature input [31]. INDUCED OF CBF EXPRESSION 1 (ICE1) is a key transcriptional regulator of the cold response [46, 79–82, 83, 84]. In addition, SNF1-RELATED PROTEIN KINASE (*KIN10)* and *COLD-RESPONSIVE15A* (*COR15A*) transcription is induced by cold [85]. To assess whether the thermal gradient table can be used to study effects of temperature dose on molecular parameters, we assessed transcript levels of *HSP70, KIN10, COR15A* and of *ICE1*. In addition we assessed PIF4 protein levels as these are knowingly enhanced at warm temperatures [18, 29, 35].

**Fig. 7:**
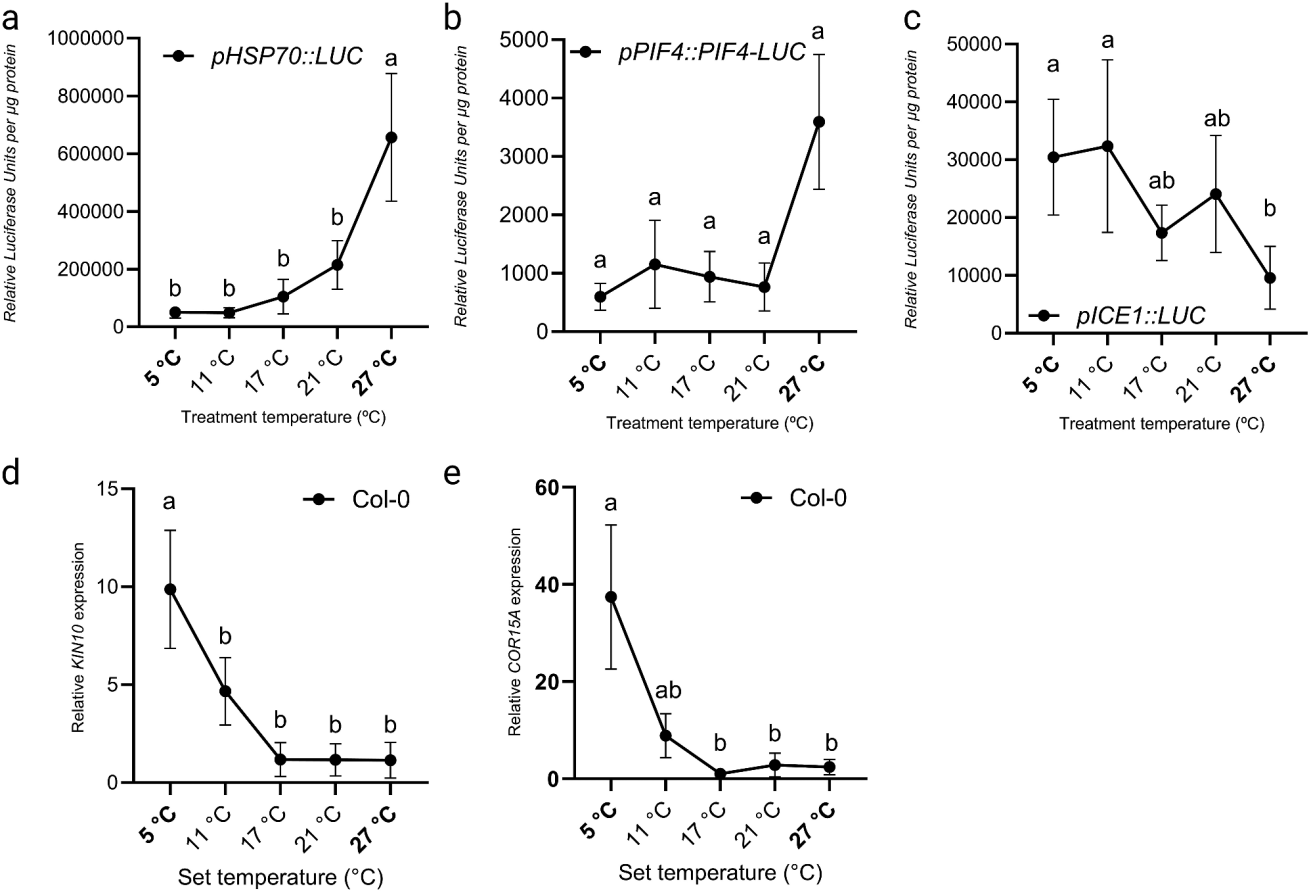
Temperature-dose effect on *HSP70*, *ICE1*,* KIN10 *and *COR15A* expression and PIF4 protein abundance. (**A**) Promoter activity of *HSP70*, (**B**) protein abundance of PIF4 and (**C**) promoter activity of *ICE1* quantified by luminescence detection of LUCIFERASE activity using transgenic lines expressing (**A**) *pHSP70::LUC*, (**B**) *pPIF4::PIF4-LUC* and (**C**) *pICE1::LUC*. The experiment was repeated five times. Relative expression of marker genes for cold acclimation (**D**) *KIN10* and (**E**) *COR15A* was determined using qRT-PCR. The experiment was repeated three times. Shown expression levels are relative to expression levels at 21 °C. Seedlings were grown on the thermal gradient with a gradient set at 5 °C to 27 °C (indicated in bold). The temperatures in-between are an average of the displayed setting on the table for each specific lane. Each sample contained approximately 30 seedlings. Error bars represent the standard error of the mean. Letters indicate statically significant differences (*P < 0.05*) as determined by one-way ANOVA with Tukey’s *post hoc* test. Error bars represent the standard error of the mean

Luciferase experiments were performed using transgenic seedlings expressing *pHSP70::LUC*,* pICE1::LUC* and *pPIF4::PIF4-LUC* grown on the thermal gradient table over a gradient set from 5 °C to 27 °C (Fig. 7A and B). As expected, *HSP70* transcript levels were enhanced when temperatures increased (Fig. 7A). This was independently confirmed by qRT-PCR experiments using Col-0 wild type seedlings that were grown on two different gradients (set from 12 °C to 32 °C and from 5 °C to 27 °C (Fig. S4A and S4B). The Luciferase experiments using seedlings expressing *pPIF4::PIF4-LUC* indicated that PIF4 protein was more abundant at 27 °C, which is in agreement with previous findings [18, 29, 35] (Fig. 7B). Although ICE1 is mostly regulated at the protein level, our analysis suggests that *ICE1* transcription is higher at low temperatures compared to control or high temperature (21 and 27 °C) (Fig. 7C). Using qRT-PCR we assessed expression levels of the cold acclimation marker genes *KIN10* and *COR15A* over a temperature gradient. As expected, expression levels of both *KIN10* and *COR15A* were significantly higher in 5 °C than in control or warm temperatures (Fig. 7D and E). Additionally, expression levels of cold-induced C-REPEAT BINDING FACTOR 2 and 3 (*CBF2* and *CBF3*) were assessed. Interestingly, these marker genes displaed a slightly increased expression in high temperatures, not in low temperatures (Fig S4C and S4D).

Taken together, the thermal gradient table is suitable for studying effects of temperature dose on the molecular (transcription and protein) level.

### Beyond Arabidopsis; use of the thermal gradient table for assessing temperature effects on lettuce and tomato

**Fig. 8:**
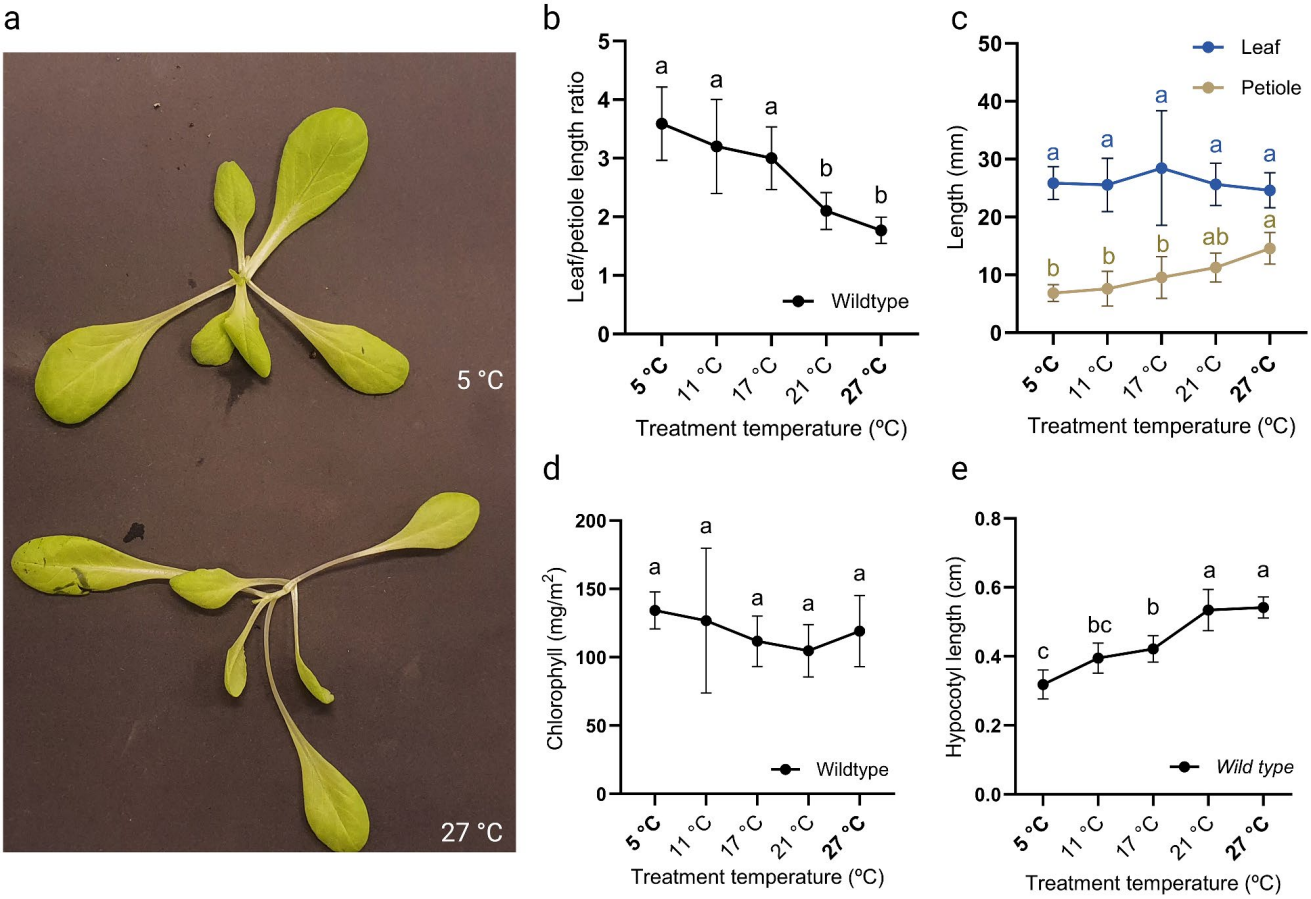
Temperature-dose effect on Butterhead lettuce. (**A**) Top photo of young butterhead lettuce plants grown at 5 °C (top) and 27 °C (bottom) on the thermal gradient table for 3 weeks. (**B**) Leaf and petiole length presented as a ratio (leaf length/petiole length) and (**C**) leaf and petiole lengths, of lettuce grown on the thermal gradient table set to a gradient of 5 °C to 27 °C (indicated in bold) in long day conditions (16-h light/ 8-h darkness). (**C**) Leaf and petiole length were measured of leaf number 3 to 5 of plants having 6 true leaves and then averaged. *N* = 5. (**D**) Chlorophyll content was measured of plants with 6 true leaves. The measurement was taken on the second youngest leaf and is an average of three measurements. *N* = 5 plants. (**E**) Hypocotyl length of seedlings measured when first true leaf emerged. Hypocotyls of 20–30 seedlings per temperature were measured when the seedlings developed the first true leaves. (**B-E**) Letters indicate statically significant differences (*P < 0.05*) within a temperature treatment as determined by one-way ANOVA with Tukey’s *post hoc* test. Error bars represent standard error of the mean

Our results illustrate that our thermal gradient table is suitable for studying temperature dose-responsiveness of *Arabidopsis thaliana*. Next, we tested if the setup can be used to assess temperature effects on commercially relevant crops. Because of its limited size and its sensitivity to temperature, butterhead lettuce (*Lactuca sativa*) was selected for this trial [86]. Lettuce seedlings were grown on the thermal gradient table on a set gradient ranging from 5 °C to 27 °C under long day photoperiod (16-h light/ 8-h darkness) until the moment the plants had developed 6 leaves. From these plants total leaf length (blade + petiole) and petiole lengths were measured and chlorophyll content was assessed. As expected, the young lettuce plants displayed internode elongation and elongated petioles at high temperatures compared to lower temperature conditions (Fig. 8A). The leaf/petiole length ratio decreased in high temperatures (Fig. 8B), due to significant petiole elongation, but no elongation of the total leaf length (petiole + blade) was detected under high temperatures (Fig. 8C). Thus, leaf blades become smaller as temperature increases (Fig. 8B). Chlorophyll content was not affected by growth temperature, similar to Arabidopsis (Fig. 8D). Lastly, lettuce hypocotyl lengths were assessed of 10 day-old seedlings subjected to a set gradient of 5 °C to 27 °C. These data indicate that lettuce hypocotyls significantly elongate in a temperature-dependent manner (Fig. 8E).

Lastly, tomato (*Solanum lycopersicum*, var. Moneymaker) seedlings were assessed using a gradient set from 12 °C to 32 °C. We observed that in agreement with literature also tomato seedlings displayed clear hypocotyl elongation in a temperature-dose dependent manner [19] (Fig. S5). Taken together, our thermal gradient table is a suitable setup for growing and assessing the effects of temperature dose on Arabidopsis and young or compact crop species.

## Discussion

Studying plant temperature dose-dependent responses over a wide temperature gradient is technically challenging since usually each temperature setting requires one cabinet or growth room. However, investigating and identifying molecular factors that regulate acclimation responses across the physiological temperature gradient is important for the development of broad-spectrum climate resilient crops [7].

Clearly, various plant traits scale with temperature dose but research assessing phenotypic and molecular changes as effect of temperature dose are relatively scarce. For instance, Liu et al., (2020) assessed Arabidopsis rosette growth, hypocotyl elongation and disease susceptibility at 16 °C, 22 °C and 28 °C in different light regimes [87]. Ibañez and colleagues showed that many phenotypes are temperature-dependent by testing plants at 16 °C, 20 °C, 24 °C and 28 °C [22]. In addition, a previous study indicated that leaf hyponasty scales with perceived temperature [5].

In this work we show that thermal gradient table setups can be used to compare phenotypic effects across the temperature spectrum, thereby overcoming the need to use multiple cabinets/growth rooms. Thermal gradient tables are readily available and thus can be swiftly implemented in research on temperature signalling and response. We show that our thermal gradient table provides a stable light and temperature environment required for in-depth analysis of plant growth along a steep temperature gradient (Fig. 1). In Arabidopsis seeds we observed that, in line with literature, temperatures above 30 °C significantly impacted germination, whereas low temperatures (< 18 °C) merely impacted germination time (Fig. 2) [66–68, 88]. Hypocotyl length positively correlated with temperature (Fig. 3), thereby confirming results of Ibañez and colleagues [22]. Using rosette-stage plants we confirmed that leaf hyponasty and petiole length increase with temperature in a dose-dependent manner and that leaf development speed is temperature dependent (Figs. 4 and 5). By using DAB staining we found significantly more ROS (hydrogen peroxide) at low temperatures compared to control and high temperatures (Fig. 6). We also observed a positive correlation between ion leakage and temperature (Fig. 6). In addition, temperature dependent transcriptional changes in *HSP70, KIN10, COR15A* and *ICE1* were found, as well as changes of PIF4 protein abundancy (Fig. 7). By growing young lettuce and tomato plants (Fig. 8 and Fig. S4) we demonstrate that our thermal gradient table is a suitable environment for growing both Arabidopsis and (young) crop plants over a temperature gradient.

Many known temperature dependent phenotypes could thus be recapitulated, but some physiological parameters, particularly chlorophyll content and stomatal conductance, did not show a clear temperature-dependency in our setup (Fig. S2), whereas previous studies showed that chlorophyll content was significantly lower in a 28/23 °C (day/night) regime compared to a 23/18 °C (day/night) regime in Arabidopsis [89]. These authors also showed that stomatal conductance and stomatal density was highest in a 25.5/20.5 °C (day/night) regime. This discrepancy might be caused by our experimental design that includes constant temperature treatment and no diurnal temperature changes. The thermal gradient table is equipped with software that allows for diurnal temperature changes, so experiments using these settings are possible.

The thermal gradient table demonstrated to be a stable and useful growth environment for growing plants over a temperature gradient but the setup has some limitations. Firstly, pots, plates or tissue culture boxes used for plant cultivation can only be heated or cooled from the bottom (aluminium plate). This is no problem for contained plates and tissue culture boxes that can be placed directly on the plate, but poses a potential issue for growing plants in pots, especially for large(r) plant species, as a vertical gradient in temperature is merely unavoidable since air temperature is not regulated. To make sure the temperature from the plate is optimally conducted to the soil (and to some extent to the air), pots need to be placed in aluminium cups. Since air humidity is not regulated, a plastic dome with holes was placed over the plants in our experiments to obtain a higher relative humidity in the direct head space of the plants. Additionally, the placement of the domes helps to control temperature-dependent soil water loss. In our experiments we used one small dome per plant (Fig. 1B). For sensitive experiments that require precise control of relative humidity, or if one e.g. wants to compare mutants with different evaporation rates, one large dome per temperature treatment (thermal gradient table row) could be considered. The necessity of the aluminium cups and plastic domes makes growing plants on the thermal gradient table relatively labour intensive and reduces the available space both in terms of growth area as in height. Additionally, without air temperature and humidity regulation, the environment outside of the table potentially has a confounding effect on the air temperature and humidity inside. To tackle this problem the thermal gradient table should be placed in an air-conditioned space with sufficient buffer. Moreover, as the lighting is provided overhead through the Plexiglass lids, the setup as an entity is not light-tight. Therefore, the table should be placed in a dark room if one wants works with daylength sensitive plants, to rule out any confounding effects of (traces of) light coming from other sources.

All sampling and phenotyping was done mostly manually and an innovation would be the instalment of e.g. overhead (low-cost Raspberry Pi) RGB camera’s that enable capturing the plant at regular intervals and enable automated image-based analysis of temperature-dose effects on plant growth, development and morphology [90]. The thermal gradient table also enables the performance of shift experiments, such as switching ambient temperature to sequentially applying low-high/high-low temperature treatments to plants, an option that deserves further exploration.

Our current set-up is large enough for experiments with seeds and seedlings on plates, but space is limited when one wants to work with plants growing in soil. This means that experiments may have to be repeated in time to obtain a suitable number of replicates per temperature treatment. Nevertheless, reaction norms obtained from one experiment across the gradient can be directly assessed as several temperature treatments can be done in parallel, but with limited replications. However, the thermal gradient table design is scalable. Constructing a larger thermal gradient setup is possible using the same design principles.

Lastly, the temperature gradient is set by adjusting the temperature of the outer two lanes. The average temperature of each row is dependent on the proportional difference between the first and last row. It is therefore not possible to set a linear gradient with equal dT between the lanes.

## Conclusions

Our data shows that thermal gradient tables can be a valuable tool to assess temperature dose effects in Arabidopsis seedlings and rosette plants, lettuce seedlings and tomato seedlings across a steep temperature gradient, in our setup potentially ranging from 5 °C to 40 °C (with a maximum dT of ~ 20 °C per experiment). Studying plant acclimation over a temperature gradient allows accessing variation that is missed when studying temperature acclimation in a binary system. Novel targets for the development of temperature resilient crops can be identified by identifying and investigating molecular factors that regulate temperature acclimation along the temperature gradient. We do invite colleagues wanting to use our thermal gradient table setup or develop/purchase their own to get in contact with us to discuss the options.

### Electronic supplementary material

Below is the link to the electronic supplementary material.

Supplementary Material 1

Supplementary Material 2

Supplementary Material 3

Supplementary Material 4

Supplementary Material 5

Supplementary Material 6

Supplementary Material 7

Supplementary Material 8

## Data Availability

No datasets were generated or analysed during the current study.

## Abbreviations

Bp: Base Pairs
CBF / *CBF*: C-REPEAT BINDING FACTOR: CBF1, CBF2 and CBF3 (protein / gene)
Col-0: Columbia-0 wild type *Arabidopsis thaliana* genotype
COR / *COR*: COLD RESPONSIVE (protein / gene)
DAB: 3,3’-Diaminobenzidine
dT: differential Temperature
EC: Evening Complex
ELF3 / *ELF3*: EARLY FLOWERING 3 (protein / gene)
H_2_O_2_: Hydrogen peroxide
*HSP70*: HEAT SHOCK PROTEIN 70 gene
ICE1 / *ICE1*: INDUCER OF CBF EXPRESSION 1 (protein / gene)
KIN10 / *KIN10*: SNF1-RELATED PROTEIN KINASE (protein/gene)
LED: Light Emitting Diode
LUC / *LUC*: LUCIFERASE (protein / gene)
MAPKs: MITOGEN ACTIVATED PROTEIN KINASES
N: Number of replicates
NS: Not Significant
PBS: 1x phosphate-buffer saline
*phyABCDE*: quadruple phytochrome ABCDE mutant
PhyB / *PhyB* / *phyB-9*: phytochrome B (protein / gene / mutant)
phyB Pfr: active phyB protein conformation (sensitive to far-red (fr) light
phyB Pr: Inactive phyB protein conformation (sensitive to fred (r) light
PIF4 /*PIF4* / *pif4-101*: PHYTOCHROME INTERACTING FACTOR 4 (protein / gene / mutant)
PIF7 / *PIF7*: PHYTOCHROME INTERACTING FACTOR 7 (protein / gene)
ROS: Reactive Oxygen Species
*UBQ10*: *UBIQUITIN10* reference gene
qRT-PCR: quantitative Real Time-Polymerase Chain Reaction
