# Supplementary material for: Using a thermal gradient table to study plant temperature signalling and response across a temperature spectrum
Source: Plant Methods. 2024 Jul 29;20:114. doi: 10.1186/s13007-024-01230-2 (PMC11285400; doi:10.1186/s13007-024-01230-2)
Supplement: Supplementary file 1 — Supplementary Material 1 [file 13007_2024_1230_MOESM1_ESM.docx]

| **Table S3. Vectors and standard parts from MoClo Basic Toolkit and MoClo Plant Parts kit used in Golden Gate cloning** | |
| --- | --- |
| **Name part** | **MoClo ID** |
| Vector level 0 CDS1stop | *pICH41308* |
| Vector level 0 PRO5U | *pICH41295* |
| Vector level 0 3U + TER | *pICH41276* |
| Vector level 1 pos. 1 | *pICH47732* |
| Vector level 1 pos. 2 | *pICH47742* |
| Vector level 2 | *pAGM4673* |
| End linker 2 | *pICH41744* |
| CD-LUC | *pICSL80001* |
| 3U + Ter-AtuNos | *pICH41421* |
| Pro-AtuNos_5U-AtuNos_CDBAR_Ter-AtuNos | *pICSL70005* |
