# Supplementary material for: Using a thermal gradient table to study plant temperature signalling and response across a temperature spectrum
Source: Plant Methods. 2024 Jul 29;20:114. doi: 10.1186/s13007-024-01230-2 (PMC11285400; doi:10.1186/s13007-024-01230-2)
Supplement: Supplementary file 2 — Supplementary Material 2 [file 13007_2024_1230_MOESM2_ESM.docx]

| **Table S1.** **Different modification made to the *pICE1* fragment compared to the native sequence to enable Golden Gate cloning.** The position numbers refer to the nucleotides (base pairs, bp) counted from the *5’*-end of the fragment, including the insertion and deletion modifications. In the modifications column the sequences are insertions on the *5’* or *3’* end of the fragment, in case of an arrow the nucleotide on the left has been substituted by the nucleotide on the right. | | |
| --- | --- | --- |
| **Argumentation** | **Position (****bp)** | **Modification** |
| Adding overhang and BpiI restriction site | 5’-end | GTCCACAGAAGACGAGGAG |
| Adding overhang and BpiI restriction site | 3’-end | AATGGAGTCTTCTGTGGTG |
| Reducing length of stretch of adenines from 13 to 11 | 326 and 327 | Removed two adenines |
| Removing BpiI internal restriction site | 551 | G → C |
| Removing BpiI internal restriction site | 954 | G → C |
| Removing BpiI internal restriction site | 1429 | C → A |
| Removing BsaI internal restriction site | 1452 | G → T |
