# Supplementary figures and images for: Using a thermal gradient table to study plant temperature signalling and response across a temperature spectrum
Source: Plant Methods. 2024 Jul 29;20:114. doi: 10.1186/s13007-024-01230-2 (PMC11285400; doi:10.1186/s13007-024-01230-2)

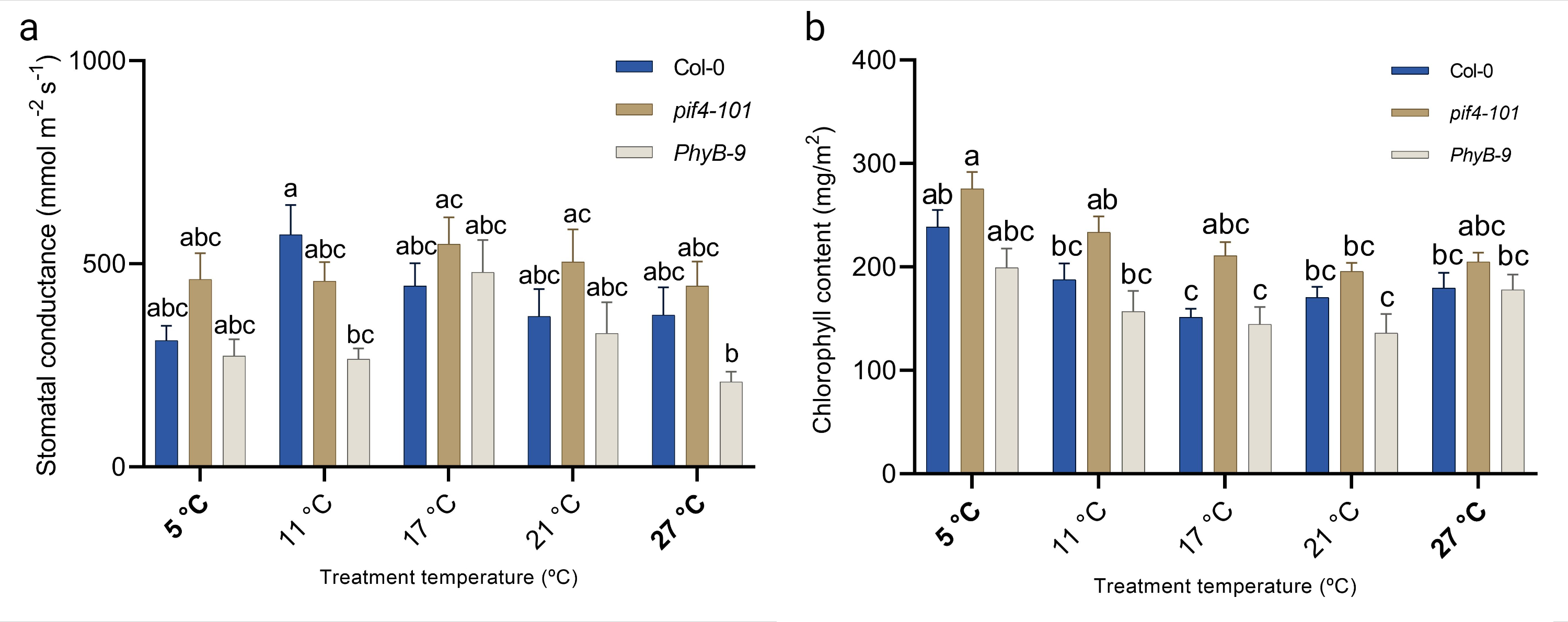

Supplement: Supplementary file 3 — Supplementary Material 3 [file 13007_2024_1230_MOESM3_ESM.jpeg]

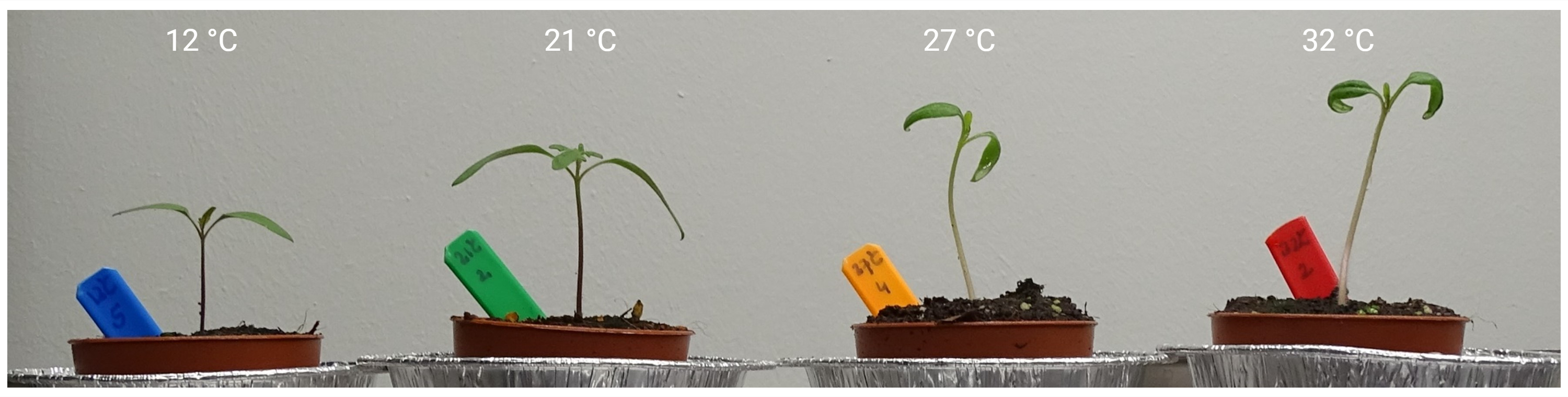

Supplement: Supplementary file 4 — Supplementary Material 4 [file 13007_2024_1230_MOESM4_ESM.jpeg]

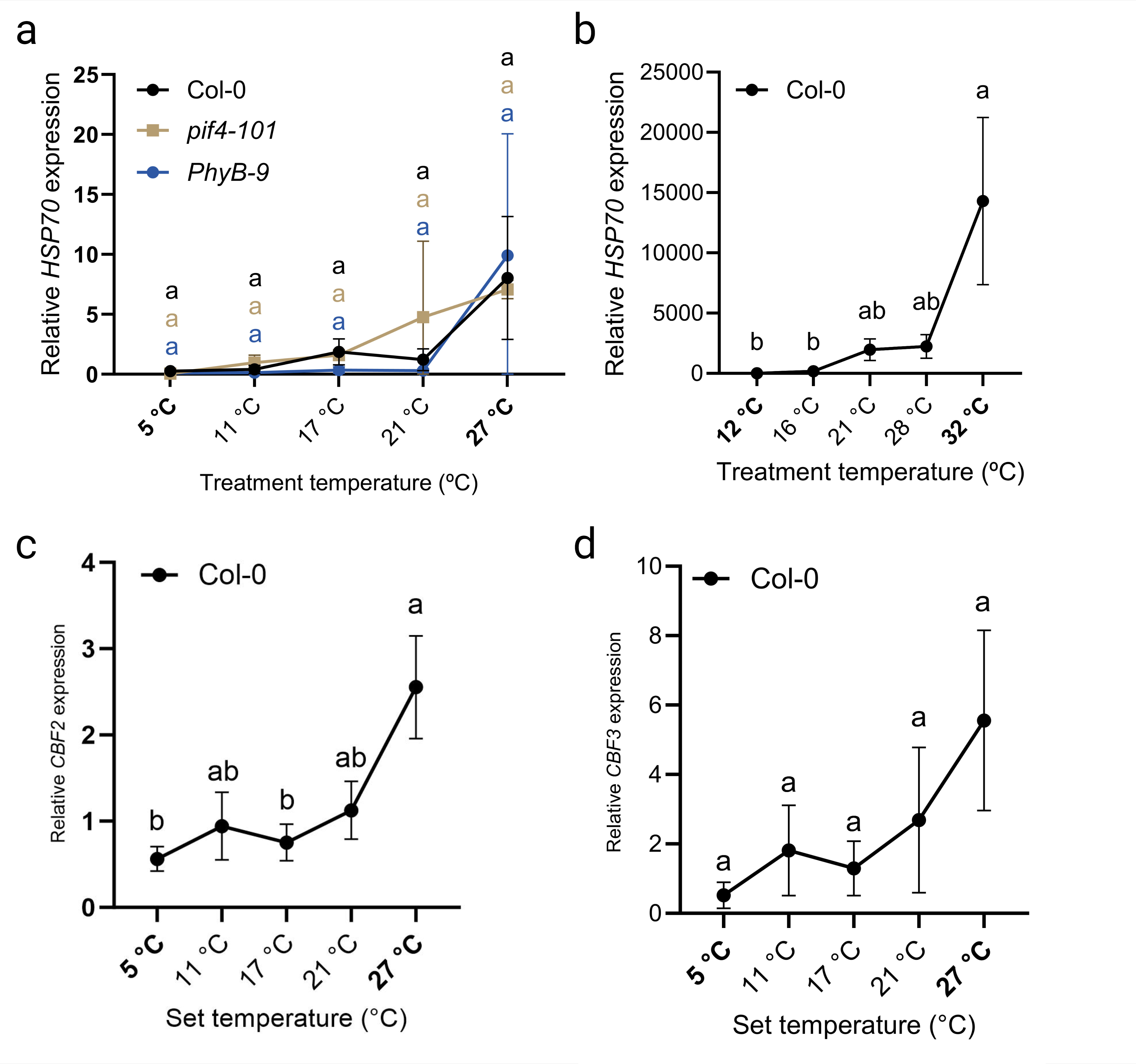

Supplement: Supplementary file 5 — Supplementary Material 5 [file 13007_2024_1230_MOESM5_ESM.jpeg]

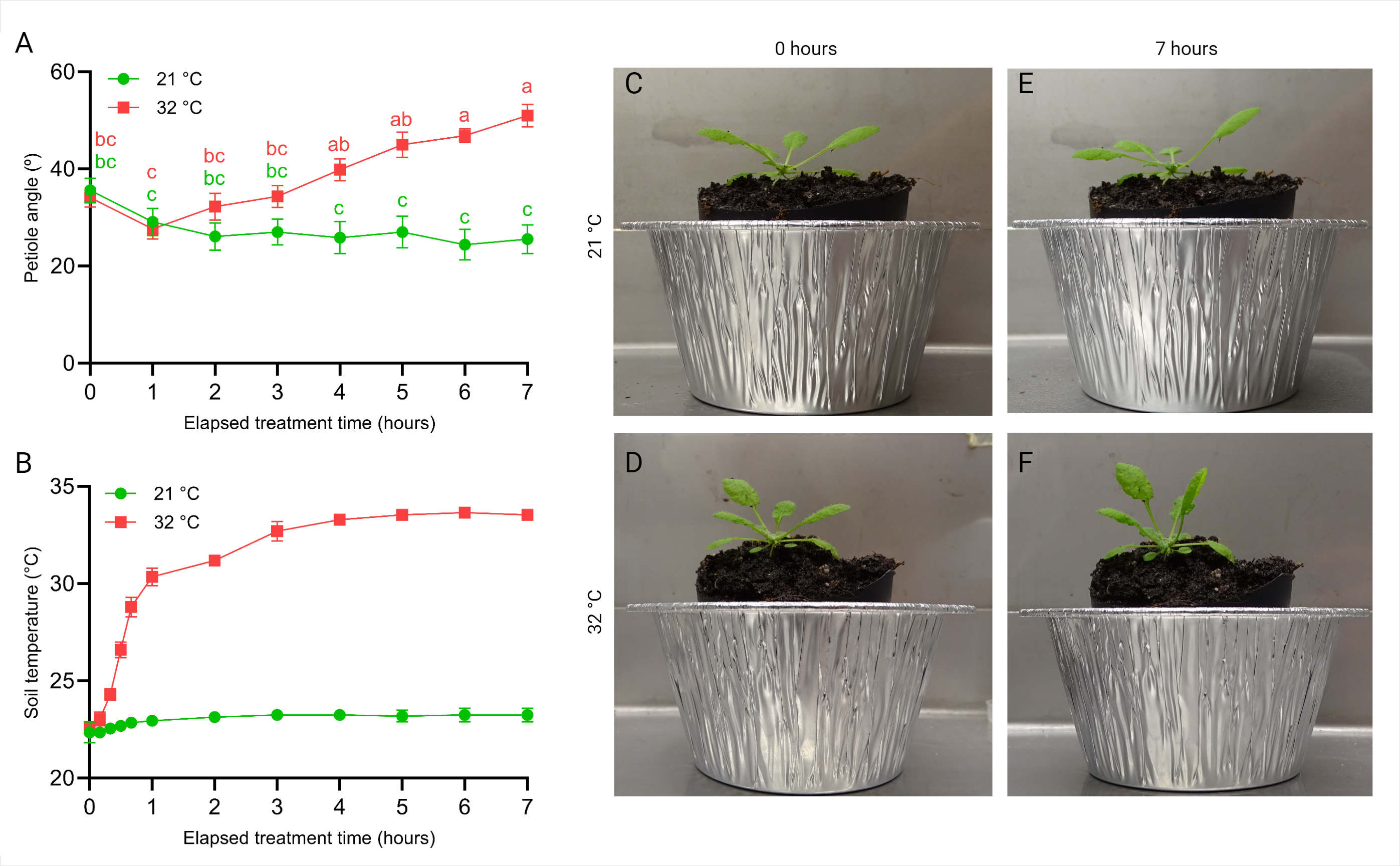

Supplement: Supplementary file 6 — Supplementary Material 6 [file 13007_2024_1230_MOESM6_ESM.jpeg]

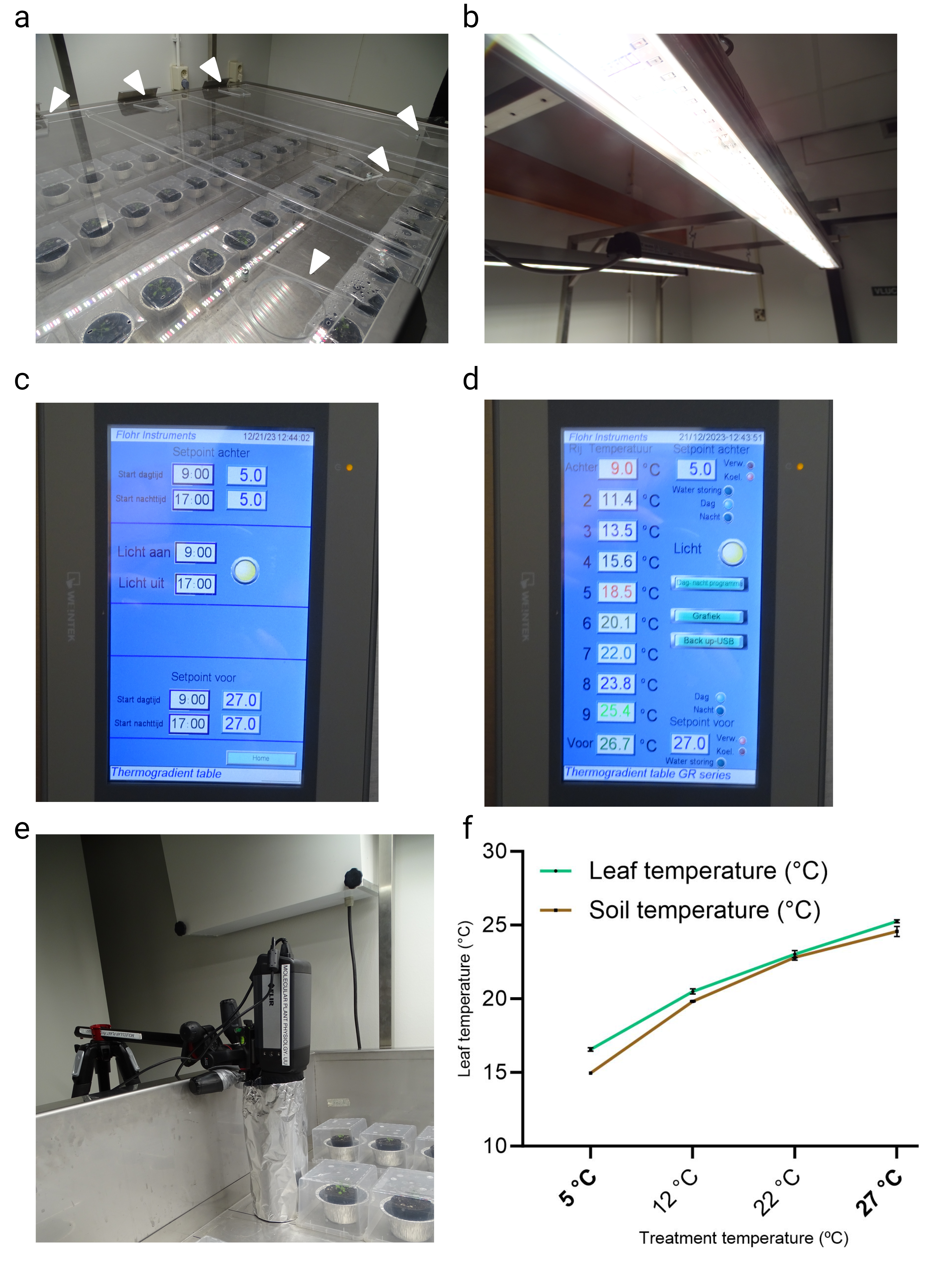

Supplement: Supplementary file 7 — Supplementary Material 7 [file 13007_2024_1230_MOESM7_ESM.jpg]
