# Supplementary material for: Using a thermal gradient table to study plant temperature signalling and response across a temperature spectrum
Source: Plant Methods. 2024 Jul 29;20:114. doi: 10.1186/s13007-024-01230-2 (PMC11285400; doi:10.1186/s13007-024-01230-2)
Supplement: Supplementary file 8 — Supplementary Material 8 [file 13007_2024_1230_MOESM8_ESM.docx]

| **Table S2. Primers used in this study.** Primers indicated with an asterisk have been published in [83]**.** | | | |  |
| --- | --- | --- | --- | --- |
| **Primer usage** | | **Forward primer** | **Reverse primer** |  |
| qRT-PCR *HSP70* (AT3G12580) | CTACCAACACCGTCTTCGATGC | | GACTCTTATCCGCTTGAACAGAGG | |
| qRT-PCR *UBQ10* ([AT4G05320](https://www.arabidopsis.org/servlets/TairObject?type=gene&id=1000639218)) | GGCCTTGTATAATCCCTGATGAATAA | | AAAGAGATAACAGGAACGGAAACATA | |
| qRT-PCR *KIN10**  (AT3G01090) | TGGAGCTGGAGCACAACA | | GACCCGAATCGCTACTTGTTC | |
| qRT-PCR *COR15A**  (AT2G42540) | GCTTCAGATTTCGTGACGGATAAAAC | | GCAAAACATTAAAGAATGTGACGGTG | |
| qRT-PCR *CBF2**  (AT4G25470) | TGACGTGTCCTTATGGAGCTA | | CTGCACTCAAAAACATTTGCA | |
| qRT-PCR *CBF3**  (AT4G25480) | GATGACGACGTATCGTTATGGA | | TACACTCGTTTCTCAGTTTTACAAAC | |
| Golden Gate/ sequencing primer/ level 0 | CTGGCCTTTTGCTCACATGT | | GTCTCATGAGCGGATACATATTTGAATG | |
| Golden Gate/ sequencing primer/ level 1 | GAACCCTGTGGTTGGCATGCACATAC | | CTGGTGGCAGGATATATTGTGGTG | |
